# Similarities and differences between MIS-C and KD: a systematic review and meta-analysis

**DOI:** 10.1186/s12969-022-00771-x

**Published:** 2022-12-05

**Authors:** Tong Tong, Xuefeng Yao, Zhe Lin, Yijing Tao, Jiawen Xu, Xiao Xu, Zhihao Fang, Zhimin Geng, Songling Fu, Wei Wang, Chunhong Xie, Yiying Zhang, Yujia Wang, Fangqi Gong

**Affiliations:** Department of Cardiology, Children’s Hospital, Zhejiang University School of Medicine, National Clinical Research Center for Child Health, No. 3333 Binsheng Road, Hangzhou, 310052 P.R. China

## Abstract

**Background:**

Multisystem inflammatory syndrome in children (MIS-C) is a new syndrome with some clinical manifestations similar to Kawasaki disease (KD), which is difficult to distinguish.

**Objective:**

The study aimed to characterize the demographic characteristics, clinical characteristics, laboratory features, cardiac complications, and treatment of MIS-C compared with KD.

**Study design:**

Studies were selected by searching the PubMed, EMBASE and so on before February 28, 2022. Statistical analyses were performed using Review Manager 5.4 software and STATA 14.0.

**Results:**

Fourteen studies with 2928 participants were included. MIS-C patients tended to be older and there was no significant difference in the sex ratio. In terms of clinical characteristics, MIS-C patients were more frequently represented with respiratory, gastrointestinal symptoms and shock. At the same time, they had a lower incidence of conjunctivitis than KD patients. MIS-C patients had lower lymphocyte counts, platelet (PLT) counts, erythrocyte sedimentation rates (ESRs), alanine transaminase (ALT), and albumin levels and had higher levels of aspartate transaminase (AST), N-terminal pro-B-type natriuretic peptide (NT-pro-BNP), troponin, C-reactive protein (CRP), D-dimer, fibrinogen, ferritin, and creatinine. MIS-C patients had a higher incidence of left ventricle (LV) dysfunction, valvular regurgitation, pericardial effusion, myocarditis, and pericarditis. The incidence of coronary artery lesion (CAL) was lower in MIS-C patients [OR (95% CI): 0.52 (0.29, 0.93), *p* =0.03], while it was similar in the acute period. MIS-C patients had higher utilization of glucocorticoids (GCs) and lower utilization of intravenous immune globulin (IVIG).

**Conclusions:**

There were specific differences between MIS-C and KD, which might assist clinicians with the accurate recognition of MIS-C and further mechanistic research.

**Supplementary Information:**

The online version contains supplementary material available at 10.1186/s12969-022-00771-x.

## Introduction

A new syndrome in children and juveniles, first described in Corona Virus Disease 2019 (COVID-19), has become a global pandemic [1–3]. The Royal College of Pediatrics and Child Health (RCPCH) defined these cases as pediatric inflammatory multisystem syndrome temporally associated with SARS-CoV-2 (PIMS-TS) [4]. The US Centers for Disease Control and Prevention (CDC) and Prevention immediately defined it as Multisystem inflammatory syndrome in children (MIS-C) [5], and the World Health Organization (WHO) named it multisystem inflammatory syndrome in children and adolescents, temporally related to COVID-19 [6]. These reports used different terminologies: MIS-C, PIMS-TS, Kawasaki-like disease or a combination of these, which we expressed uniformly with MIS-C. In the pediatric population, MIS-C is noted to be a novel syndrome that is temporally associated with previous exposure to severe acute respiratory syndrome coronavirus 2 (SARS-CoV-2), which fully or partially meets the diagnostic criteria of KD [2, 3]. Patients may present with high fever, lymphadenopathy, pleomorphic rash, conjunctivitis, mucosal pathology changes, and coronary artery dilation [7]. However, some differences in these two diseases have been identified, and they may be derived from different pathophysiologies, which have not yet been clarified. There is no clear indication of whether these cases are caused by SARS-COV-2 infection, distinct clinical entities, or simple coincidences arising from a seasonal increase in the incidence of KD. This systematic review and meta-analysis discussed the similarities and differences between MIS-C associated with COVID-19 and KD based on demographic characteristics, clinical characteristics, laboratory features, cardiac complications, and treatment to help distinguish patients with MIS-C from those with KD and provide deeper insight into them.

## Methods

Our study was registered on the International Prospective Register of Systematic Reviews [8], and the protocol can be obtained at https://www.crd.york.ac.uk/prospero/display_record.php? ID=CRD42022315554 (registration number: CRD42022315554). This meta-analysis was performed according to the guidelines of the Preferred Reporting Items for Systematic Reviews and Meta-analyses (PRISMA) Statement.

### Search strategy

We searched and retrieved data from PubMed (MEDLINE), EMBASE, Web of Science, the Cochrane Library, Wan Fang and China National Knowledge Infrastructure. All publications until 30 February 2022 were searched without any restriction of countries. The search string was built as follows: ('multisystem inflammatory syndrome' OR 'pediatric inflammatory multisystem syndrome' OR 'pediatric multisystem inflammatory syndrome' OR PIMS-TS OR PMIS OR MIS-C) AND ('mucocutaneous lymph node syndrome' OR 'Kawasaki disease' OR 'Kawasaki syndrome'). Both English and non-English literature was identified.

### Study selection

Two independent reviewers screened the titles and abstracts using the search strategies mentioned above to identify and assess potentially eligible studies. Any disagreement would be resolved by discussion until consensus was reached or by consulting a third author. All the studies met the inclusion and exclusion criteria described herein.

### Inclusion criteria

Eligible studies met PICOS criteria (participants, interventions, comparators, outcomes, and study design). The included study types were observational studies investigating the difference between MIS-C and KD. MIS-C patients are diagnosed by the definition of CDC, WHO, or RCPCH [4–6]. KD patients are defined by the 2017 American Heart Association (AHA) criteria [2]. Patients with KD were deliberately enrolled from the pre-pandemic period to avoid diagnostic confusion.

### Exclusion criteria

Duplicate publications, conference abstracts, reviews, editorials, comments, meta-analyses, letters, notes, and studies that did not report on prespecified outcomes were excluded.

### Data extraction and quality assessment

The following data were extracted: title, author, year of publication, sample size, country, original inclusion criteria, relevant data, and terms of quality assessment. The NOS score was used to assess the quality of observational studies based on the three areas of selection, comparability, and outcome/exposure [9]. The studies with an NOS score >6 were categorized as high quality, and those with an NOS score of 4–6 were classified as medium quality. Articles of poor quality (NOS score of 0–3) were excluded.

### Statistical analysis

The statistical analysis was performed using Review Manager 5.4 software and STATA 14.0. Dichotomous data were summarized by ORs and 95% CIs. For the continuous data, WMDs were the first choice in the pooled effect sizes, but for different units of laboratory values or significant deviations from the mean, SMDs were calculated. Statistical significance was assumed at a P value of less than 0.05. The Q test and I^2^ values were used to evaluate the heterogeneity across studies. We defined I^2^ values of 25% or less, near 50%, and near 75% or greater as low, moderate, and high degrees of heterogeneity, respectively. If the I^2^ value was <50%, a FEM was used; otherwise, a REM was chosen. If the I^2^ value was >75%, statistical heterogeneity was apparent and could be eliminated by adjusting the clinical confounding factors, subgroup analysis, and sensitivity analysis. Publication bias was assessed with Egger’s test, and each dot represents one study in the meta-analysis.

### Subgroup and sensitivity analysis

Subgroup analysis was conducted based on the experimental group of MIS-C-KD or MIS-C patients. MIS-C-KD patients met the criteria for both MIS-C and KD. Furthermore, sensitivity analysis was performed by eliminating each study at one time to evaluate the influence of each trial on the primary outcome and the robustness of the results.

## Result

### Search results and characteristics of included studies

**Fig. 1:**
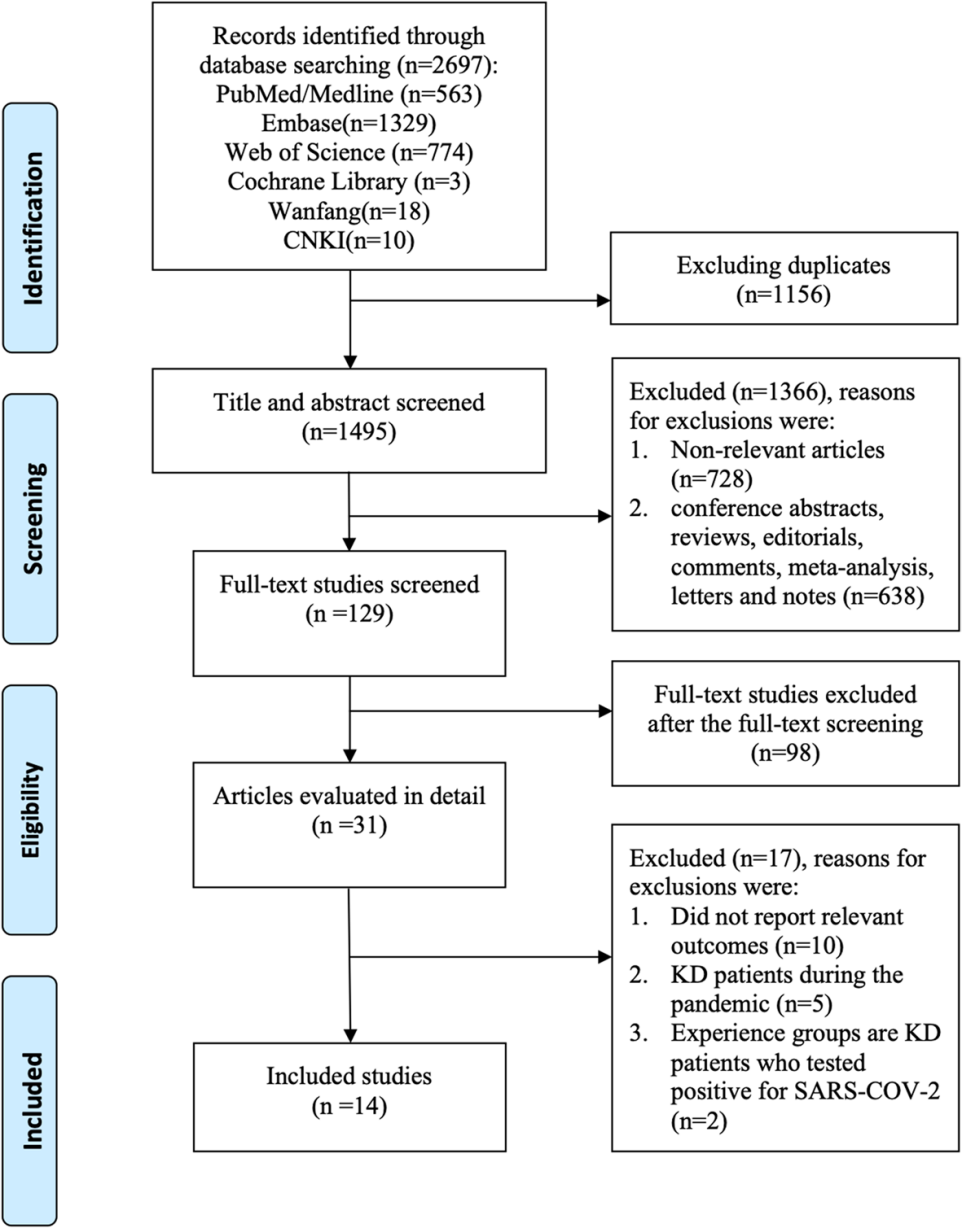
Flow diagram of the literature search and study selection

We identified 2697 citations in the PubMed, EMBASE, Web of Science, Cochrane Library, Wan Fang and CNKI databases. We excluded 2522 articles on the basis of duplications, screening titles, and published abstracts. An additional 115 articles were excluded after reviewing the full texts. Finally, a total of 14 studies met our selection criteria [10–20]. The flowchart for screening studies is shown in Fig. 1. Data from 2928 patients (2127 KD patients, 956 MIS-C patients and 20 MIS-C-KD patients) were included in this meta-analysis. Among the eligible studies, 12 studies compared MIS-C and KD, and 2 studies compared MIS-C-KD and KD. For the quality assessment, 2 studies were of moderate quality, and 12 studies were of high quality. The characteristics of all included studies are summarized in Table 1.

**Table 1:** Characteristics of included studies

First author	year	country	Study period	Intervention group	inclusion standards	number	Control group	number	NOS score
Carbajal, R [10].	2020	France	MIS-C: Apr.1-Jul. 15,2020; KD: jan.1,2018-Jul.15 2020.	MIS-C	WHO CDC RCOPC	7	KD	40	7
Cattalini, M [21].	2021	Italy	MIS-C: Feb.1-May31, 2020; KD:2000-2019	PIMS-TS	RCOPC	53	KD	96	7
Cherqaoui, B [22].	2021	France	MIS-C: 2020; KD:2011-2014	PIMS	NA	404	KD	425	6
Consiglio, C. R [20].	2020	Italy and Sweden	MIS-C:Mar.17-May15 2020; KD: Mar. 2017-May 2019	MIS-C	WHO	13	KD	28	6
Corwin, D. J [23].	2020	US	MIS-C: Mar.1-May 15 2020; KD: 2019	MIS-C-KD	CDC	8	KD	15	8
Esteve-Sole, A [19].	2021	Spain	MIS-C: Apr. 23–Jun. 5, 2020; KD: 2016–2019	MIS-C	WHO RCOPC	14	KD	14	8
Felsenstein, S [18].	2021	UK	MIS-C:Mar.2020-Mar.2021; KD: Feb. 2005-Feb. 2020	PIMS-TS	CDC RCOPC	34	KD	27	8
Gaitonde, M [17].	2020	US	MIS-C: Mar.-Jun.2020; KD : Jan. - Jun. 2019	MIS-C	CDC	12	KD	12	7
Godfred, S [16].	2022	US	MIS-C:Mar.16, 2020- Feb.21, 2021; KD: Jan.2019- Dec. 2019	MIS-C	CDC	233	KD	101	7
Kostik, M. M [15].	2021	Russia	MIS-C: May 2020-Apr.2021; KD:Sep. 2010-Feb. 2021	MIS-C	WHO	72	KD	147	7
Lee, P. Y [14].	2020	US	MIS-C:March to June 2020; KD: May 2019- Feb. 2020	MIS-C	WHO CDC RCOPC	28	KD	40	7
Matsubara, D [13].	2020	US	MIS-C: Apr. 10-Jun. 7, 2020; KD:Jan.2019 - Dec.2019	MIS-C	WHO CDC	28	KD	20	8
Mohsin, S. S [12].	2021	Pakistan	MIS-C: May-Aug. 31,2020; KD: in the last five years prior to this pandemic	MIS-C-KD	WHO*	12	KD	30	7
Whittaker, E [11].	2020	UK	MIS-C: Mar. 23-May 16, 2020; KD:2002–2019	PIMS-TS	WHO CDC RCOPC	58	KD	1132	7

NOTE: NOS: Newcastle–Ottawa scale; * defined by WHO except for requirement for SARS-CoV-2 exposure contact history

### Meta-analysis of demographic characteristics

**Fig. 2:**
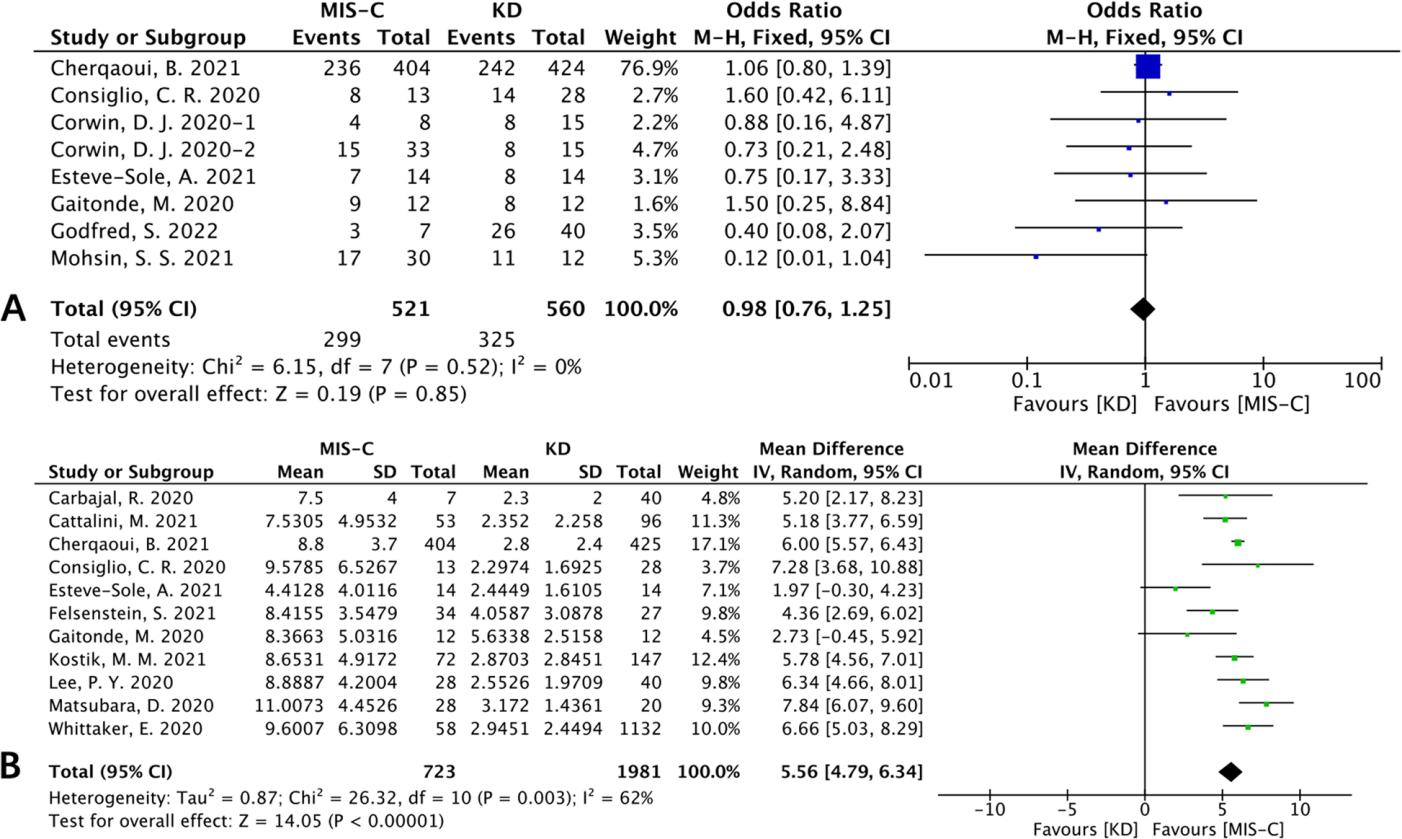
Forest plots of laboratory biochemistry parameters. **A** Forest plot for sex ratio between the MIS-C group and the KD group. **B** Forest plot for age between the MIS-C group and the KD group

Pooled data from our meta-analysis that included eight studies showed that there was no significant difference in the sex ratio between MIS-C and KD patients (Fig. 2A). However, regarding age, eleven studies were included in this meta-analysis, and we concluded that MIS-C was common in older children and teenagers (Fig. 2B).

### Meta-analysis of clinical characteristics

**Fig. 3:**
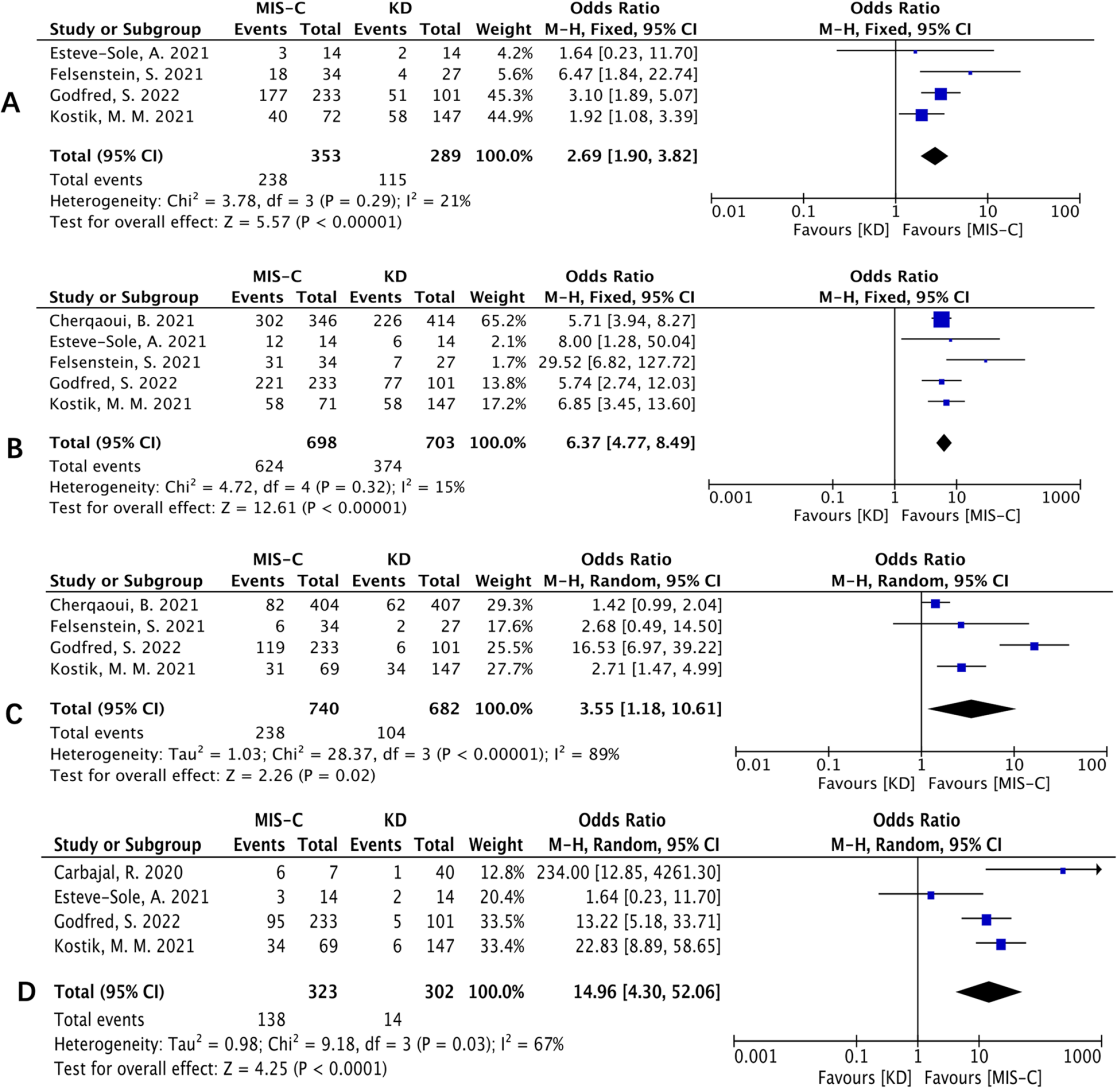
Forest plots of clinical characteristics. **A** Forest plot for respiratory symptoms. **B** Forest plot for gastrointestinal symptoms. **C** Forest plot for neurological symptoms. **D** Forest plot for shock

Four studies were included in the analysis of the respiratory symptoms between MIS-C patients and KD patients, which showed that respiratory symptoms were more common in MIS-C patients [OR (95% CI): 2.69 (1.90, 3.82), *p*<0.00001; Fig. 3A]. Pooled data from our meta-analysis that included five studies showed that gastrointestinal symptoms were more common in MIS-C patients than in KD patients [OR (95% CI): 6.37 (4.77, 8.49), *p*<0.00001; Fig. 3B]. Three studies showed that the incidence of arthralgia or arthritis was not significantly different between MIS-C and KD patients (OR (95% CI): 0.84 (0.49, 1.43), *p*=0.52; heterogeneity: *p* =0.84, I^2^ = 0%). We found that neurological symptoms were more common in MIS-C patients than in KD patients [OR (95% CI): 3.55 (1.18, 10.61), *p*=0.02; Fig. 3C], and there was high heterogeneity (I^2^ =89%). Four studies reported the incidence of shock, and the pooled data showed a high rate of shock in MIS-C patients [OR (95% CI): 14.96 (4.30, 52.06), *p*<0.0001; Fig. 3D].

As for characteristic clinical manifestations of KD, we found that MIS-C patients had a lower incidence of rash [OR (95%CI): 0.26 (0.10, 0.68), *p*=0.006], conjunctivitis [OR (95%CI): 0.29 (0.12, 0.71), *p*=0.007], oral changes [OR (95%CI): 0.18 (0.04, 0.74), *p*=0.02] and cervical lymphadenopathy [OR (95%CI): 0.37 (0.15, 0.96), *p*=0.04] than KD patients. No significant difference was found in the rate of extremity changes (OR (95% CI): 0.40 (0.15, 1.04), *p*=0.06). However, high levels of heterogeneity were found (I^2^ > 75%).

### Meta-analysis of laboratory features

**Fig. 4:**
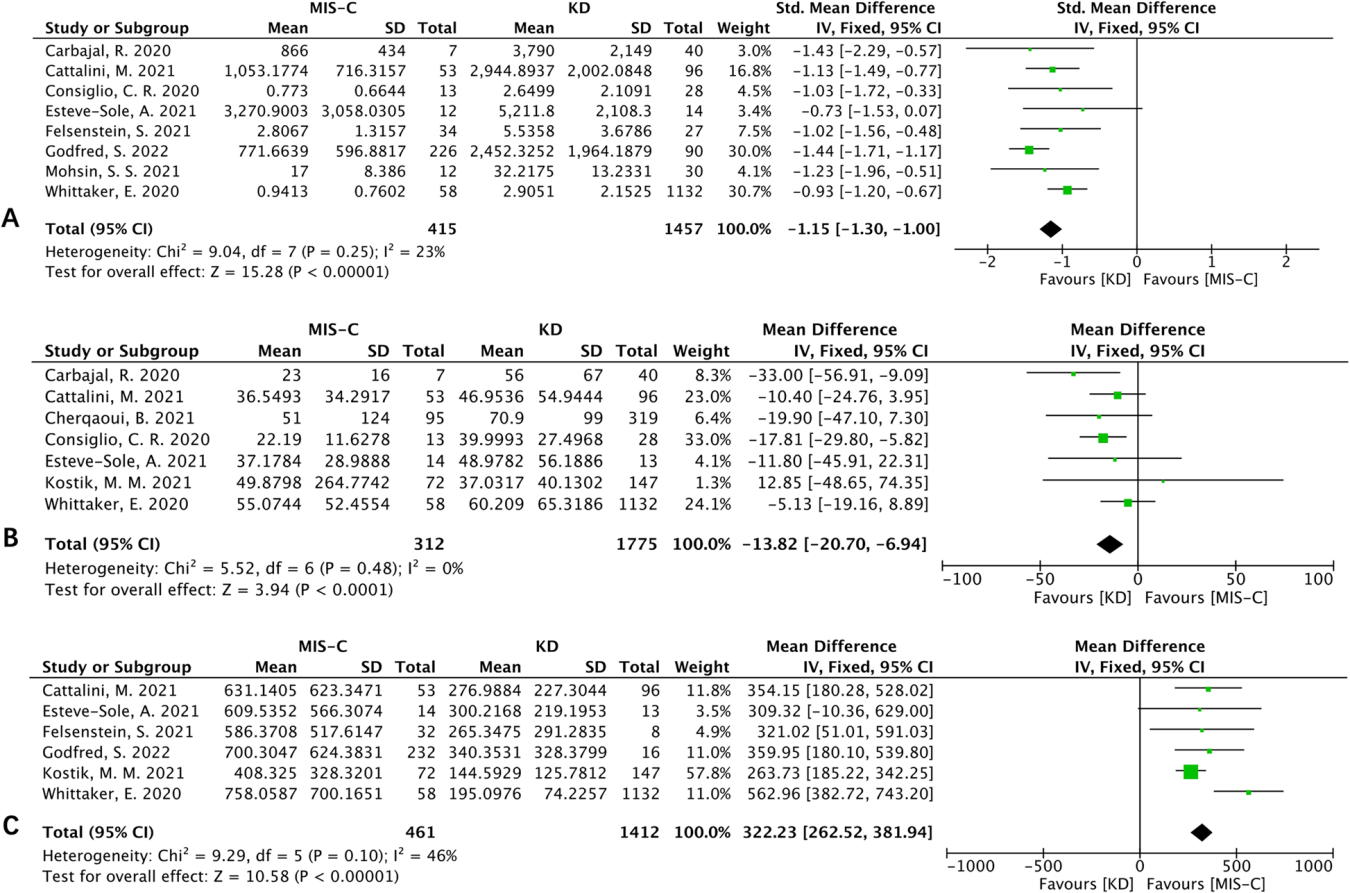
Forest plots of laboratory features I. **A** Forest plot for lymphocyte count. **B** Forest plot for ALT. **C** Forest plot for ferritin

For laboratory features, our meta-analysis indicated that MIS-C patients had lower lymphocyte count levels [SMD (95% CI): −1.15 (−1.30, -1.00), *p* < 0.00001; Fig. 4A], lower ALT levels [MD (95% CI): -13.82 (-20.7, -6.94), *p* < 0.0001; Fig. 4B], lower ESR levels [SMD (95% CI): −0.25 (−0.45, −0.05), *p* =0.02; heterogeneity: *p* = 0.19, I^2^ = 38%], higher D-dimer levels [MD (95% CI): 1.25 (0.78, 1.71), *p* < 0.00001; heterogeneity: p = 0.03, I^2^ = 67%; Fig. 4D], higher fibrinogen levels [MD (95% CI): 0.71 (0.26, 1.17), *p* =0.002; heterogeneity: *p* = 0.46, I^2^ = 0%], higher ferritin levels [MD (95% CI): 322.23 (262.52, 381.94), p < 0.00001; Fig. 4C], higher creatinine levels [SMD (95% CI): 1.68 (0.77, 2.58), *p* = 0.0003; heterogeneity: *p* = 0.06, I^2^ = 72%], higher AST levels [SMD (95% CI): 0.34 (0.15, 0.54), p = 0.0006; heterogeneity: *p* = 0.65, I^2^ = 0%] than KD patients. No significant difference was found in Neutrophil count levels between the two groups (Fig. S1). Four studies reported NT-proBNP with high heterogeneity, and the pooled data showed that MIS-C patients had higher NT-proBNP levels than KD patients [SMD (95% CI): 1.45 (0.72, 2.18), *p* =0.0001; Fig. S2]. The pooled data of six studies showed no significant difference in sodium levels between the two diseases with high heterogeneity (Fig. S4). Three studies reported troponin levels, all of which indicated that MIS-C patients had higher troponin levels. However, there was high heterogeneity, and the pooled data showed that MIS-C patients had higher troponin levels than KD patients [MD (95% CI): 155.48 (64.54, 246.41), *p* =0.01; Fig. S5]. Seven studies reported albumin and the pooled data showed MIS-C patients had lower albumin levels with a high level of heterogeneity [MD (95% CI): -6.46 (-10.82, -2.09), *p* =0.004; Fig. S6].

**Fig. 5:**
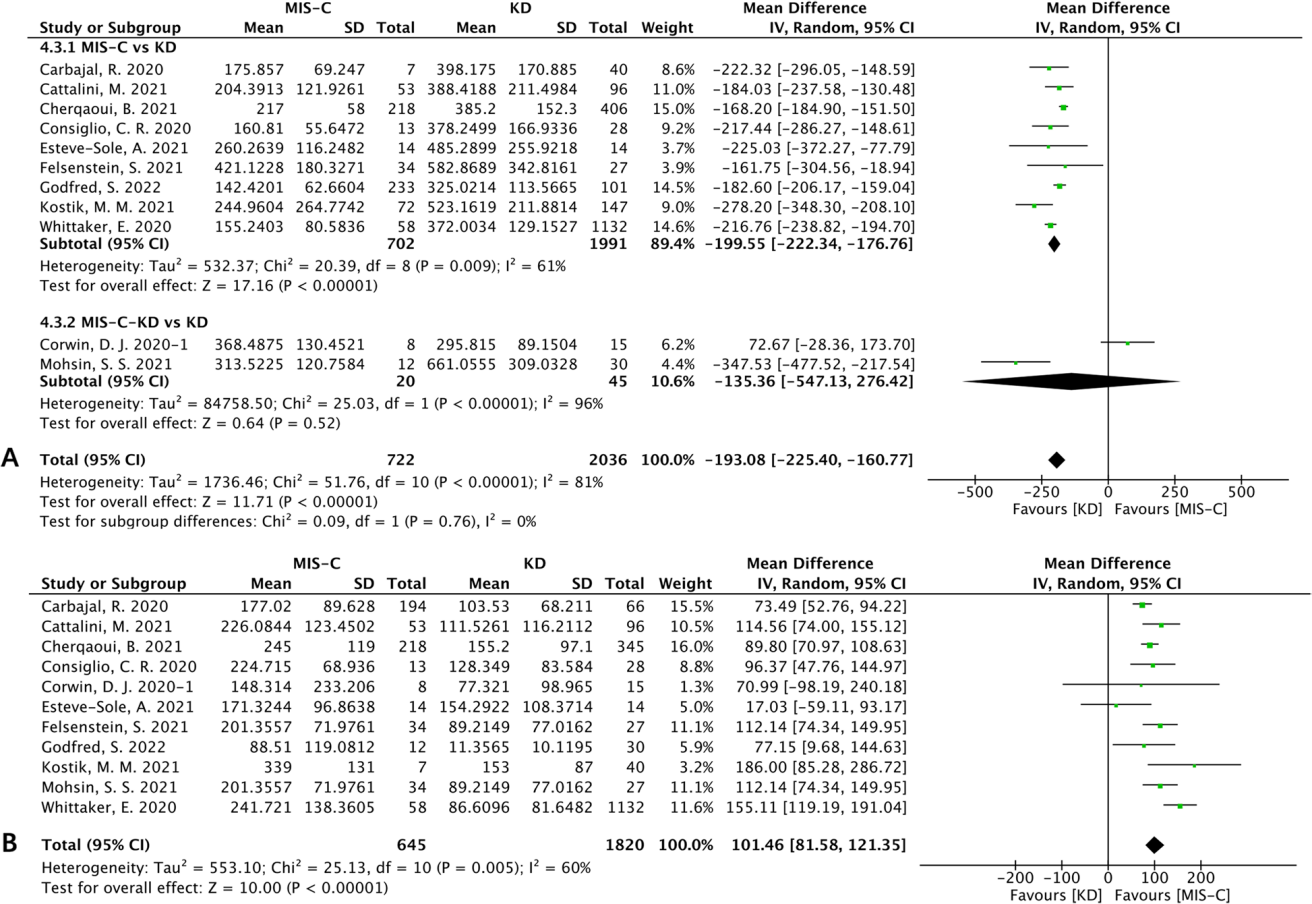
Forest plots of laboratory features II. **A** Forest plot for platelet count. **B** Forest plot for CRP

Eleven studies reported PLT, and there was high heterogeneity (I^2^>75%). Therefore, we conducted a subgroup analysis on the basis of the experimental group of MIS-C-KD or MIS-C patients, which showed that the PLT levels were higher in MIS-C patients than in KD patients [MD (95% CI): -199.55 (-222.34, -176.76), *p* < 0.00001; Fig. 5A]. The meta-analysis showed a higher CRP level in MIS-C patients [MD (95% CI): 101.46 (81.58, 121.35), *p* < 0.00001; Fig. 5B]. Then, a subgroup analysis was performed based on the experimental groups of MIS-C-KD or MIS-C patients and showed that they both had higher CRP levels than KD patients.

### Meta-analysis of cardiac complications

**Fig. 6:**
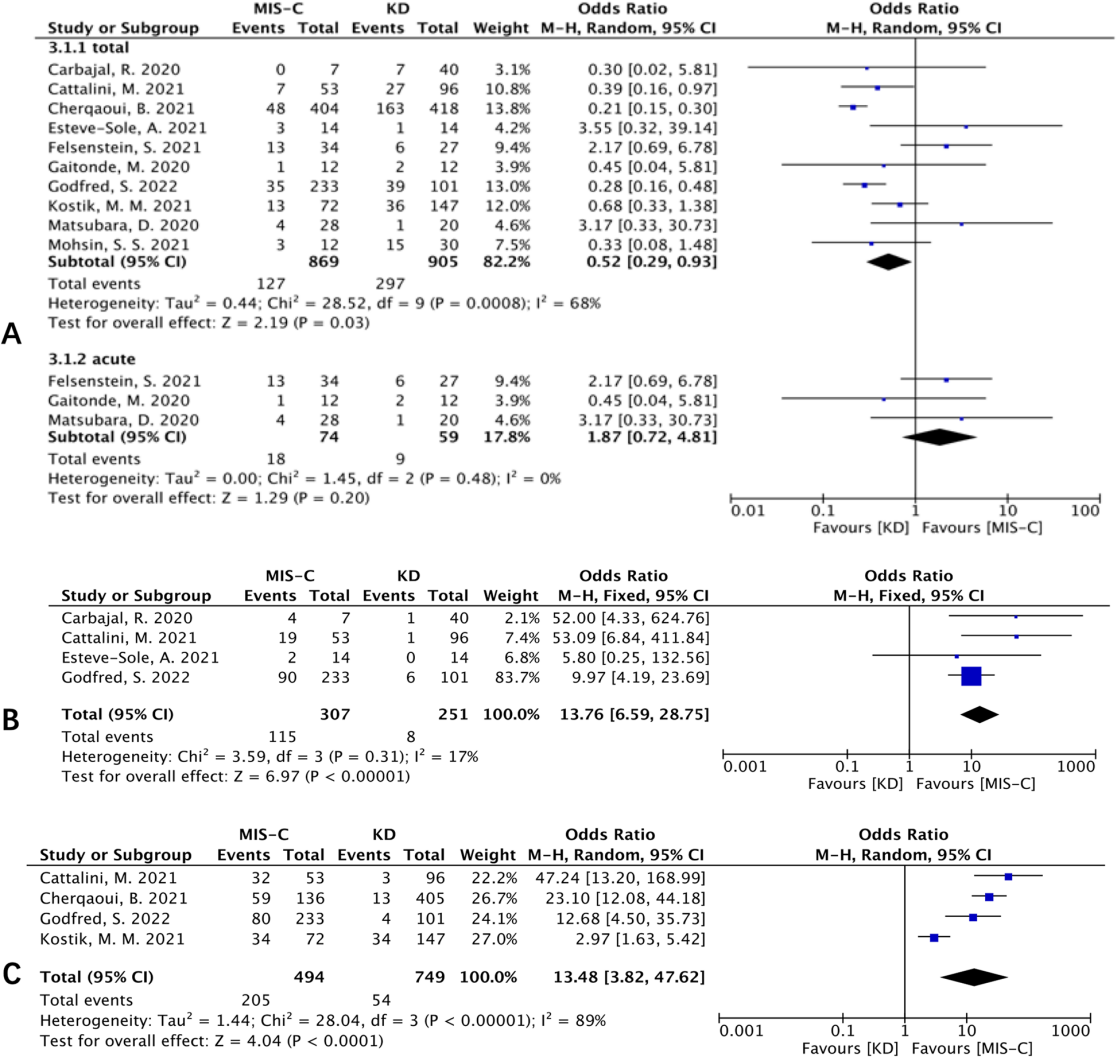
Forest plots of cardiac complications. **A** Forest plot for CAL. **B** Forest plot for decreased LV function. **C** Forest plot for myocarditis

Ten studies were included in the analysis of CAL, including cardiac dilations and aneurysms, between MIS-C patients and KD patients. The meta-analysis showed that the incidence of CAL was lower in MIS-C patients [OR (95% CI): 0.52 (0.29, 0.93), *p* =0.03; Fig. 6A]. Then, we compared the incidence of CAL between the two diseases in the acute phase of KD. As a result, there was no significant difference in the incidence of CAL in the acute phase between MIS-C and KD patients (Fig. 6A). The LV dysfunction outcome from 4 studies indicated that it was more common in MIS-C patients than in KD patients [OR (95% CI): 13.76 (6.59, 28.75), *p* <0.00001; Fig. 6B]. Additionally, the pooled outcome from five studies showed that MIS-C patients had a higher incidence of valvular regurgitation [OR (95% CI): 5.93 (3.38, 10.41), *p* <0.00001; heterogeneity: *p* =0.40, I^2^ = 1%], and pericardial effusion [OR (95% CI): 2.71 (1.20, 6.12), *p* =0.01; heterogeneity: *p* =0.02, I2 = 66%]. No significant difference was found in abnormal electrocardiography between the two groups (OR (95% CI): 20.78 (0.87, 495.02), *p* =0.06; heterogeneity: *p* =0.15, I^2^ = 52%). Four studies reported myocarditis, all of which showed a high incidence of myocarditis in MIS-C patients with high heterogeneity. The pooled data showed that MIS-C patients had a higher incidence of myocarditis than KD patients [OR (95% CI): 13.48 (3.82, 47.62), *p*<0.0001; Fig. 6C]. Three studies reported pericarditis; although the heterogeneity was high, the pooled data showed that MIS-C patients had a higher incidence of pericarditis than KD patients (OR (95% CI): 4.46 (1.31, 15.18), *p*=0.02; heterogeneity: *p* =0.002, I^2^ = 84%).

### Meta-analysis of treatment

**Fig. 7:**
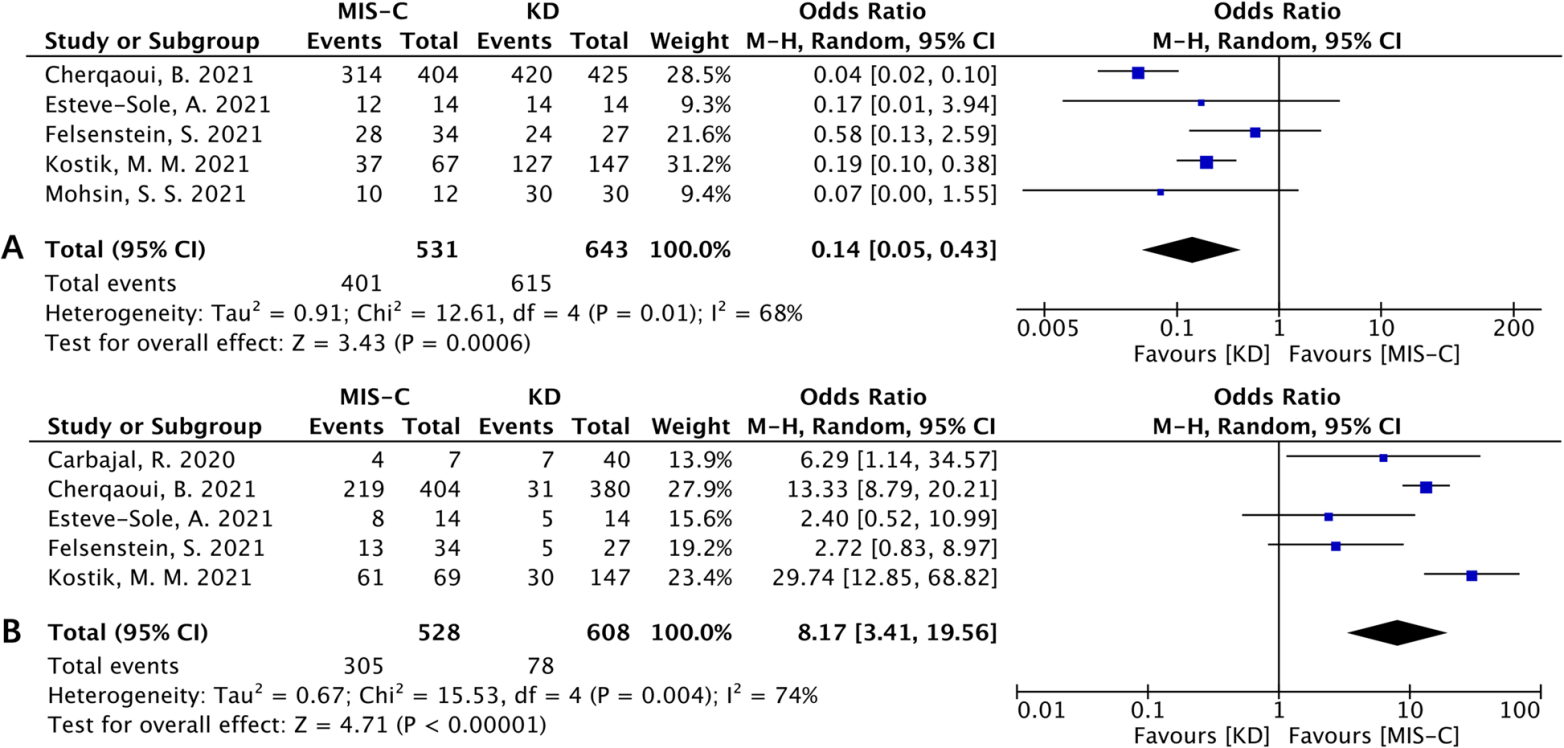
Forest plots of treatment. **A** Forest plot for the use of IVIG between the MIS-C group and the KD group. **B** Forest plot for the use of GC between the MIS-C group and the KD group

Regarding treatment, the use of IVIG was less common in MIS-C patients than in KD patients [OR (95% CI): 0.14 (0.05, 0.43), *p*=0.0006; Fig. 7A], while the use of glucocorticoids (GC) was more common in MIS-C patients [OR (95% CI): 8.17 (3.41, 19.56), *p*<0.00001; Fig. 7B].

### Publication bias

We calculated the publication bias in the comparison including more than ten studies. The Egger’s publication bias plot showed no obvious asymmetry, without significant publication bias (Fig. S14-16).

## Discussion

MIS-C was first described during the global pandemic of COVID-19, which is closely related to COVID-19 infection [1, 24]. In addition to the CDC, case definitions have also been published by the WHO and the RCPCH, although the RCPCH does not require evidence of prior exposure to SARS-CoV-2 [4]. In epidemiological, clinical, and immunological investigations, MIS-C has been shown to share many of the same phenotypic features with KD. There is still a debate that MIS-C may be considered a separate clinical entity or a more severe form of KD [25]. It may be difficult to distinguish these patients from those with classical KD who lack evidence of previous SARS-CoV-2 exposure. Although anti-SARS-CoV-2 IgM or IgG has been useful in distinguishing MIS-C early in the pandemic, the specificity of seropositivity as a diagnostic marker for MIS-C decreases because population immunity builds from ongoing exposure or subsequent vaccination. In light of the ongoing outbreak of COVID-19, it is imperative to improve our understanding of this new disease, particularly developing more accurate diagnostic criteria that clearly differentiate this entity from KD to adopt adequate and appropriate therapy. Kostik M. M et al. [15] create a Kawasaki/MIS-C differentiation score, allowing discrimination of MIS-C and KD. Five criteria, CRP >11 mg/dl (18 points), D-dimer >607 ng/ml (27 points), age >5 years (30 points), thrombocytopenia (25 points), and GI involvement (28 points), were included in the score. The summa >55 points allowed us to discriminate MIS-C from KD with a sensitivity of 87.5% and specificity of 89.1%. To distinguish patients with MIS-C who go on to critical illness from those with classic KD, we conducted a systematic review and meta-analysis to show the differences between these two diseases.

Clinical symptoms of respiratory, gastrointestinal, and neurologic symptoms are more common in MIS-C patients. The heterogeneity was high in analyzing neurological symptoms (Fig. S8). To exclude the influence of a single study on the results of the meta-analysis, sensitivity analysis was adopted. As a result, regardless of which study remained excluded, the results showed a higher incidence of neurologic symptoms in MIS-C patients. These clinical differences may result from the presence of autoantibodies in MIS-C patients against not only endothelial but also gastrointestinal and immunocompetent cells. Approximately 65% of MIS-C patients admitted to ICU had CNS involvement [15]. On the other hands, neurological involvement was observed in 5.1% (80/1582) of KD patients with a positive prognosis, and it was not a risk factor for CALs [26]. Regarding the typical manifestations of KD, including rash, conjunctivitis, oral changes, and cervical lymphadenopathy, the incidence in KD patients was significantly higher than that in MIS-C patients, despite the high heterogeneity. We performed a sensitivity analysis (Fig. S9-13) and found that, whichever study excluded the pooled data, all concluded that MIS-C patients had a lower incidence of conjunctivitis (Fig. S10). However, the other results were not stable. Therefore, further well-designed studies are needed to explore the differences between these clinical KD symptoms to better identify the two diseases. In the meantime, a higher proportion of MIS-C patients presented with shock compared with KD, which means MIS-C is more severe than KD, requiring prompt identification.

Regarding cardiac complications, the most prevalent cardiac symptom of MIS-C is LV dysfunction, which is followed by CAL and electric conduction anomalies [27]. Most markedly, our study showed a greater degree of overall LV dysfunction in the MIS-C group. However, many studies reported that LV function normalized in most patients after treatment. If no treatment is given, coronary dilation and aneurysms are the main complications (26%-40%) in KD. When treated promptly with IVIG, they were reduced to 3% to 6% [2]. However, in MIS-C, the incidence of CAL varies significantly between reports. Our meta-analysis revealed that the incidence of CAL was lower in MIS-C patients. Only a small number of studies clearly recorded when CAA occurred. Three of ten studies reported the acute phase, and the pooled data showed no significant difference. In the Felsenstein, S. In 2021 [18], 6 KD patients (*n*=27) and 13 MIS-C patients (*n*=34) presented with coronary dilation or aneurysms within eight weeks. However, within three months, only 1 MIS-C patient and 6 KD patients remained abnormal (*p*=0.01), which meant that the incidence of CAL was higher in KD patients during the subacute stage. In most cases of MIS-C, abnormalities improve rapidly after treatment, while they tend to remain in KD: during the subacute stage, CALs were documented in more than 40% of patients with KD but only in 12.5% of MIS-C patients [28]. It is worth noting that the better coronary outcomes in MIS-C could have been affected by the more intensive immunomodulatory treatment. During KD, neutrophils infiltrate the vessel wall and cause necrotizing arteritis [29]. In addition, subacute vasculitis may occur in association with myofibroblastic proliferation and result in late stenoses [30]. The causes of coronary dilatation in MIS-C are far from being well understood. The cause of CAL in MIS-C may be associated with fever and circulating inflammatory mediators or may be related to inflammation as well as disruption of the arterial wall, as is seen in KD. As transient coronary artery dilatation can also be seen in febrile children with noncardiac infections, systemic inflammation and cytokine storms may contribute to transient coronary and cardiac pathology. Fortunately, in almost all instances of coronary dilatation in MIS-C patients, changes normalized within 2–3 months [18], which was in striking contrast to KD. Even significant coronary dilatation in MIS-C had the potential to normalize. This may further support the hypothesis that systemic inflammation and a cytokine storm contribute to cardiac pathology in MIS-C. We also found that MIS-C patients more frequently had myocarditis with a high level of heterogeneity. After sensitivity analysis was adopted, we did not detect any significant impact from any single study and confirmed the direction of the results in the present study. An autopsy report of a fatal case with MIS-C found substantial evidence of myocarditis pericarditis endocarditis with inflammatory cell infiltration [31]. A fulminant lymphocytic myocarditis secondary to an imbalanced proinflammatory response was observed in the endomyocardial biopsy of a 19-year-old MIS-C patient [32]. Variability in clinical presentation in patients may be due to different mechanisms. In addition, we can conclude from our analysis that other cardiac complications, such as valvular regurgitation, pericardial effusion, abnormal electrocardiography, and pericarditis, are significantly higher in MIS-C patients. Only a few studies have reported posthospital discharge follow-up data, and most cardiac abnormalities were reported to have resolved at discharge or on follow-up, except for a few patients [33]. Nevertheless, it remains unknown what the long-term outcomes of MIS-C will be. Follow-up studies and long-term cardiac surveillance are needed to monitor late effects on cardiac function and ascertain the development of coronary abnormalities.

A lower lymphocyte count may indicate an association with COVID-19, a process that is probably controlled by the initiation of homing factors and widespread apoptosis/cell death by IL-6 and Fas-FasL interactions [34]. However, Fabi M. et al. discovered that MIS-C patients had similar lymphocyte subpopulations compared to KD patients [28]. In KD, the fundamental pathogenesis is thought to be mediated by immune complexes, which activate inflammatory cells (including monocytes and neutrophils), leading to the recruitment of platelets and thrombocytosis [35]. In contrast, in MIS-C, mediators are secreted to eradicate the virus, which inadvertently suppress bone marrow function and activate platelets, eventually making the patients thrombocytopenic [36]. This may explain why platelets were significantly lower in MIS-C patients than in KD patients. Elevated CRP, D-dimer, fibrinogen, and ferritin levels indicated that MIS-C presented with a more severe inflammatory state than KD. However, ESR showed a lower level in MIS-C patients, which might result from the universal application of IVIG in KD patients. The inflammatory markers showed more severe systemic inflammation or stronger inflammatory reactions in MIS-C patients than in KD patients. D-dimer and ferritin showed higher levels in MIS-C patients, similar to macrophage activation syndrome. D-dimer reflects the increased production of thrombin and dissolution of fibrin, and fibrinogen is an important substrate for thrombosis. A high level of D-dimer and fibrinogen correlates with disease severity in adults with COVID-19. They may increase the risk for venous and arterial thrombosis. Both arterial and venous thrombosis are theoretically at risk in patients with MIS-C because of endothelial injury and abnormal platelet activation and coagulation. However, such a relationship is not found in MIS-C [37]. Another characteristic of patients with MIS-C is a marked increase in biochemical markers of cardiac injury, including troponin and NT-pro-BNP. There were high levels of heterogeneity in the analysis of troponin and NT-proBNP. After the sensitivity analysis (Fig. S3), we found that the result was stable. In KD, myocardial stunning or edema is the main finding without ischemic damage and with limited cell necrosis, as evidenced by the mild to moderate elevation of troponin and the high level of BNP [38, 39]. In terms of biochemistry, MIS-C patients had lower levels of albumin, ALT and higher levels of creatinine, and AST than KD patients. Seven studies reported albumin, although a high level of heterogeneity existed. The sensitivity analysis (Fig. S7) showed that there was no single study that influenced the outcome. As a result of inflammation, the increased permeability of capillaries, increasing expression of vascular endothelial growth factor, increasing escape, increasing distribution volume, shortened half-life, and decreasing total mass of albumin are considered to be the associated pathophysiology of hypoalbuminemia.

IVIG is now the standard of care for KD and can alleviate symptoms and stabilize patients within hours of administration [40]. Current MIS-C treatments rely on empirical methods and are not sufficiently supported by evidence and IVIG was deemed to be a first-line therapy to treat MIS-C [41]. Nevertheless, IVIG alone may be less effective in inducing defervescence in MIS-C than IVIG with corticosteroids [42]. Management of MIS-C should involve multidisciplinary care with intensive care, cardiology, infectious disease, and rheumatology specialists. Treatment strategies include symptomatic and supportive therapy, along with immunomodulatory treatment. In the meantime, antiplatelet therapy with low-dose aspirin should be taken into consideration in patients who meet the criteria for KD, have coronary artery changes, or have other risk factors for thrombosis [43]. Because MIS-C is more serious than KD, most patients are hospitalized in the intensive care unit, where 25–100% need inotropic support or vasopressors [42].

### Study Limitations

Our study has several limitations that should be considered. First, some of the outcomes may still show residual heterogeneity, even though sensitivity analyses and subgroup analyses were conducted. Therefore, the results must be interpreted cautiously. Second, the selected studies were mainly observational, which increased the possibility of heterogeneity and limited the reliability of the results. Furthermore, several of the studies lacked sufficient sample sizes, which may reduce statistical power and influence heterogeneity. Overall, based on the findings of this meta-analysis, larger-scale studies with more extended follow-up periods should be carried out to investigate more critical information.

## Conclusion

Our meta-analysis confirms that MIS-C has specific characteristics compared to KD, including being more common in older patients, more frequent respiratory and gastrointestinal involvement, shock, more cardiac complications (myocarditis, LV dysfunction, valvular regurgitation, pericardial effusion and pericarditis), fewer conjunctivitis symptoms, higher levels of inflammatory markers (CRP, D-dimer, fibrinogen and ferritin), myocardial injury markers (NT-proBNP, troponin, AST) and creatine, and lower levels of lymphocyte count, PLT count, albumin, ALT and ESR. The incidence of CAL was lower in MIS-C patients, while it was similar in the acute period of the disease. As long as the COVID-19 pandemic continues, clinicians should keep a high degree of suspicion for this severe form of KD.

## Supplementary Information

**Additional file 1: Figure S1.** Neutrophil count in the meta-analysis of six studies using the random-effects model. **Figure S2.** NT-proBNP in the meta-analysis of four studies using the random-effects model. **Figure S3.** Sensitivity analysis of NT-pro-BNP. **Figure S4.** Sodium in the meta-analysis of six studies using the random-effects model. **Figure S5.** Troponin in the meta-analysis of six studies using the random-effects model. **Figure S6.** Albumin in the meta-analysis of six studies using the random-effects model. **Figure S7.** Sensitivity analysis of albumin. **Figure S8.** Sensitivity analysis of neurological symptoms. **Figure S9.** Sensitivity analysis of cervical lymphadenopathy. **Figure S10.** Sensitivity analysis of conjunctivitis. **Figure S11.** Sensitivity analysis of oral changes. **Figure S12.** Sensitivity analysis of rash. **Figure S13.** Sensitivity analysis of extremity changes. **Figure S14.** Egger’s publication bias plots for the assessment of potential publication bias in the analysis of age. **Figure S15.** Egger’s publication bias plots for the assessment of potential publication bias in the analysis of CRP. **Figure S16.** Egger’s publication bias plots for the assessment of potential publication bias in the analysis of platelet count.

## Data Availability

The data that support the fndings of this study are available on request from the corresponding authors, FQ Gong.

## Abbreviations

MIS-C: Multisystem inflammatory syndrome in children
KD: Kawasaki disease
COVID-19: Coronavirus disease 2019
RCPCH: Royal College of Pediatrics and Child Health
PIMS-TS: Pediatric multisystem inflammatory syndrome temporally associated with COVID-19
CDC: Centers for Disease Control
WHO: World Health Organization
PLT: Platelet
ESRs: Erythrocyte sedimentation rates
ALT: Alanine transaminase
AST: Aspartate transaminase
NT-pro-BNP: N-terminal pro-B-type natriuretic peptide
CRP: C-reactive protein
LV: Left ventricle
CAL: Coronary artery lesion
GCs: Glucocorticoids
IVIG: Intravenous immune globulin
NOS: Newcastle-Ottawa Scale
ORs: Odds ratios
CIs: Confidence intervals
WMDs: Weighted mean differences
SMDs: Standard mean differences
FEM: Fixed-effect model
REM: Random-effect model
