# Supplementary material for: Similarities and differences between MIS-C and KD: a systematic review and meta-analysis
Source: Pediatr Rheumatol Online J. 2022 Dec 5;20:112. doi: 10.1186/s12969-022-00771-x (PMC9721002; doi:10.1186/s12969-022-00771-x)
Supplement: Supplementary file 1 — Additional file 1: Figure S1. Neutrophil count in the meta-analysis of six studies using the random-effects model. Figure S2. NT-proBNP in the meta-analysis of four studies using the random-effects model. Figure S3. Sensitivity analysis of NT-pro-BNP. Figure S4. Sodium in the meta-analysis of six studies using the random-effects model. Figure S5. Troponin in the meta-analysis of six studies using the random-effects model. Figure S6. Albumin in the meta-analysis of six studies using the random-effects model. Figure S7. Sensitivity analysis of albumin. Figure S8. Sensitivity analysis of neurological symptoms. Figure S9. Sensitivity analysis of cervical lymphadenopathy. Figure S10. Sensitivity analysis of conjunctivitis. Figure S11. Sensitivity analysis of oral changes. Figure S12. Sensitivity analysis of rash. Figure S13. Sensitivity analysis of extremity changes. Figure S14. Egger’s publication bias plots for the assessment of potential publication bias in the analysis of age. Figure S15. Egger’s publication bias plots for the assessment of potential publication bias in the analysis of CRP. Figure S16. Egger’s publication bias plots for the assessment of potential publication bias in the analysis of platelet count. [file 12969_2022_771_MOESM1_ESM.pdf]

	MIS-C			KD				Std. Mean Difference
**Study or Subgroup**	**Mean**	**SD**	**Total**	**Mean**	**SD**	**Total**	**Weight**	**IV, Random, 95% CI**
Carbajal, R. 2020	9,783	7,205	7	9,572	4,948	40	10.5%	0.04 [−0.76, 0.84]
Cattalini, M. 2021	10,004.1228	5,905.7943	53	10,457.1778	6,608.3852	96	18.9%	−0.07 [−0.41, 0.26]
Consiglio, C. R. 2020	5.954	2.6578	13	9.7144	4.8432	28	12.3%	−0.86 [−1.55, −0.17]
Esteve-Sole, A. 2021	7,465.8896	4,977.8833	14	10,275.3799	3,919.4533	13	10.9%	−0.61 [−1.38, 0.17]
Felsenstein, S. 2021	12.4692	6.3463	34	11.4645	6.9659	27	15.5%	0.15 [−0.36, 0.66]
Mohsin, S. S. 2021	74.6337	10.9018	12	56.713	15.5684	30	11.7%	1.22 [0.49, 1.94]
Whittaker, E. 2020	14.0601	6.842	58	14.0601	6.842	1132	20.3%	0.00 [−0.26, 0.26]
**Total (95% CI)**			**191**			**1366**	**100.0%**	**−0.02 [−0.37, 0.34]**

Heterogeneity: $Tau^2 = 0.14$; $Chi^2 = 19.75$, df = 6 (P = 0.003); $I^2 = 70\%$

Test for overall effect: Z = 0.08 (P = 0.93)

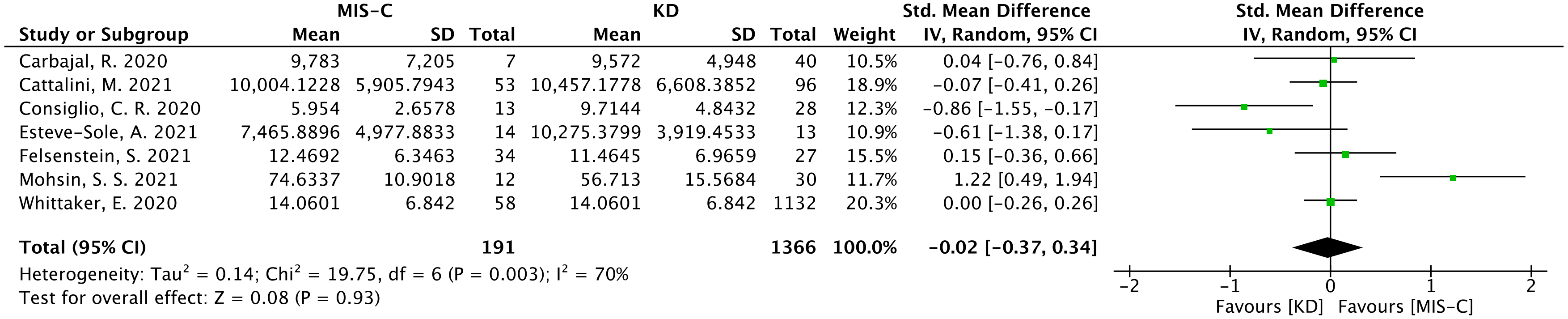

Fig. S1. Neutrophil count in the meta-analysis of six studies using the random-effects model.

Test for overall effect, SMD (95% CI): -0.02 (-0.37, 0.34), p =0.93

Study or Subgroup	MIS-C Mean	MIS-C SD	MIS-C Total	KD Mean	KD SD	KD Total	Weight	Std. Mean Difference IV, Random, 95% CI
Cattalini, M. 2021	1,132.4876	787.1852	53	254.4158	202.4665	96	26.5%	1.76 [1.37, 2.15]
Esteve-Sole, A. 2021	605.54713	659.92092	14	216.48752	198.27401	13	21.5%	0.76 [−0.02, 1.55]
Godfred, S. 2022	793.29747	1,026.31702	174	19.5415	33.30529	13	24.4%	0.78 [0.21, 1.35]
Whittaker, E. 2020	4,019.8495	7,886.55	58	52.2055	66.8031	1132	27.5%	2.29 [2.01, 2.57]
**Total (95% CI)**			**299**			**1254**	**100.0%**	**1.45 [0.72, 2.18]**

Heterogeneity: $Tau^2 = 0.49$; $Chi^2 = 30.90$, df = 3 ($P < 0.00001$); $I^2 = 90\%$

Test for overall effect: Z = 3.89 (P = 0.0001)

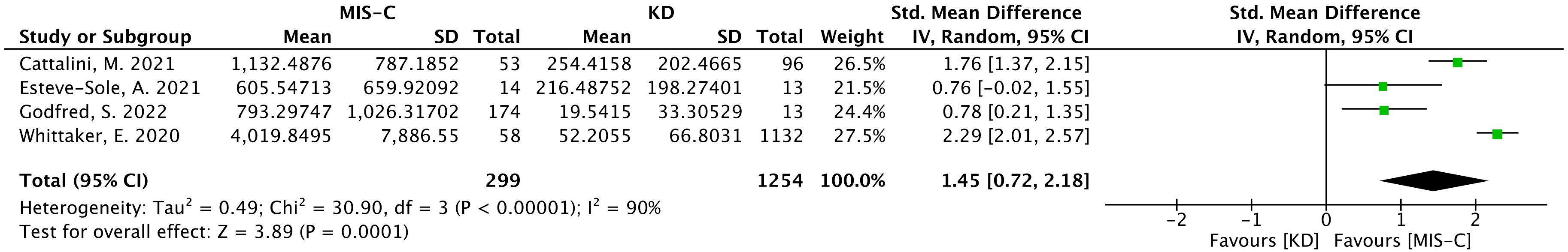

Fig. S2. NT-proBNP in the meta-analysis of four studies using the random-effects model.

Test for overall effect, SMD (95% CI): 1.45(0.72, 2.18), p =0.0001

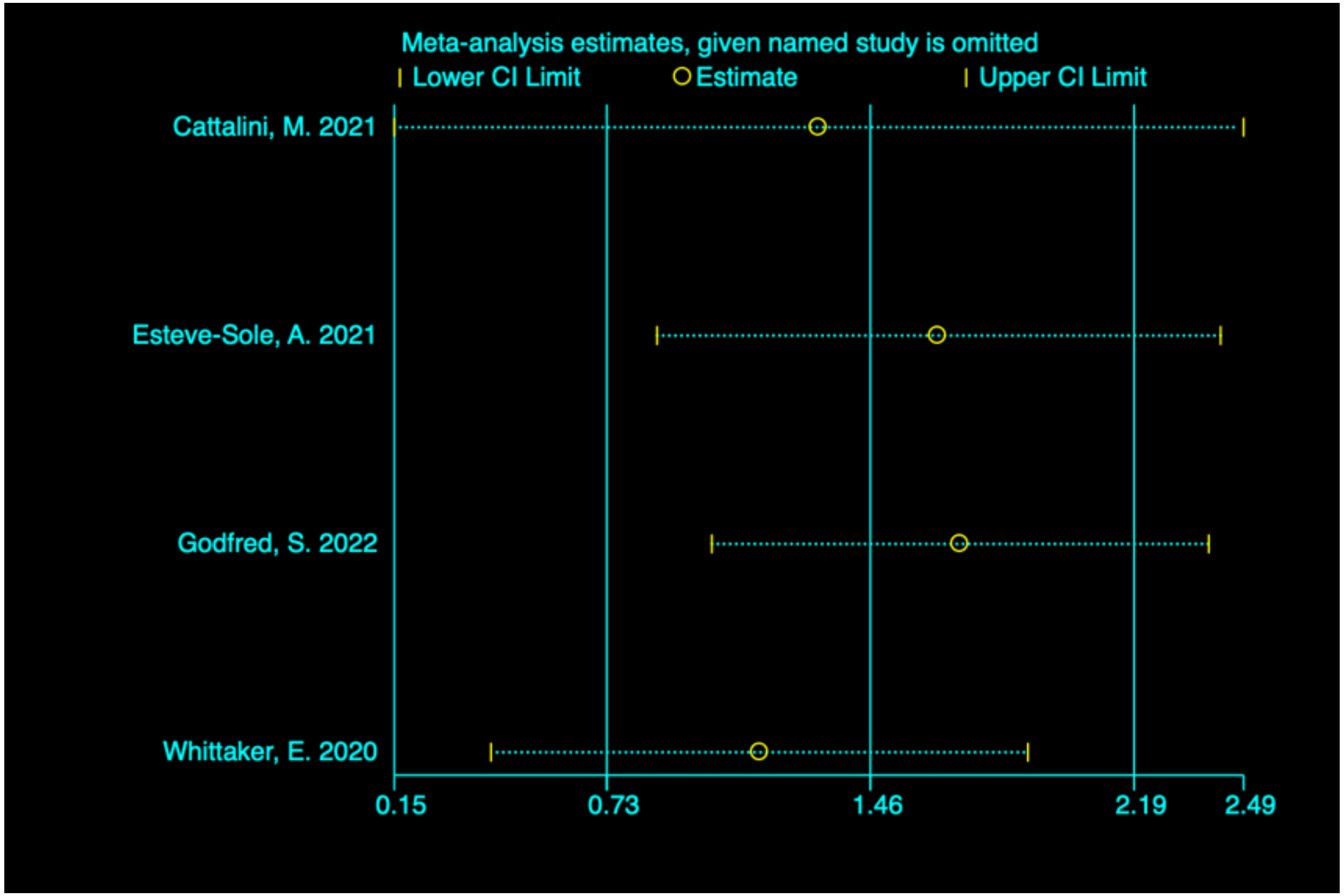

Fig. S3. Sensitivity analysis of NT-pro-BNP.

Not any single study was detected to incur undue weight in the analysis.

Study or Subgroup	MIS-C Mean	MIS-C SD	MIS-C Total	KD Mean	KD SD	KD Total	Weight	Mean Difference IV, Random, 95% CI
Carbajal, R. 2020	130	4.2	7	134	3	40	18.3%	-4.00 [-7.25, -0.75]
Cherqaoui, B. 2021	129.9	3.6	53	134.5	0.4	356	25.7%	-4.60 [-5.57, -3.63]
Consiglio, C. R. 2020	134.1205	3.5714	13	135.643	2.3435	28	22.3%	-1.52 [-3.65, 0.60]
Corwin, D. J. 2020-1	139.1181	11.6156	8	134	1.6358	15	6.9%	5.12 [-2.97, 13.21]
Esteve-Sole, A. 2021	136.5	12.3561	14	136.1825	8.7208	13	7.0%	0.32 [-7.71, 8.34]
Felsenstein, S. 2021	134.7115	4.6436	34	134	6.2615	27	19.8%	0.71 [-2.12, 3.54]
**Total (95% CI)**			**129**			**479**	**100.0%**	**-1.74 [-4.21, 0.73]**

Heterogeneity: $Tau^2 = 5.93$; $Chi^2 = 22.07$, df = 5 (P = 0.0005); $I^2 = 77\%$
Test for overall effect: Z = 1.38 (P = 0.17)

Mean Difference
IV, Random, 95% CI

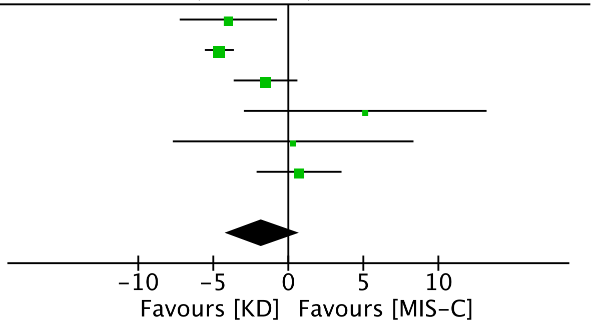

Fig. S4. Sodium in the meta-analysis of six studies using the random-effects model.

Test for overall effect, MD (95% CI): -1.74(-4.21, 0.73), p =0.17

Study or Subgroup	MIS-C Mean	MIS-C SD	MIS-C Total	KD Mean	KD SD	KD Total	Weight	Mean Difference IV, Random, 95% CI
Cattalini, M. 2021	315.1306	451.1265	53	10.6886	15.8059	96	24.8%	304.44 [182.95, 425.94]
Godfred, S. 2022	230.16	168.6	230	123.84	86.59	9	37.1%	106.32 [45.70, 166.94]
Whittaker, E. 2020	119.9128	217.4237	58	13.5017	7.4226	1132	38.1%	106.41 [50.45, 162.37]
**Total (95% CI)**			**341**			**1237**	**100.0%**	**155.48 [64.54, 246.41]**

Heterogeneity: $Tau^2 = 4839.11$; $Chi^2 = 9.16$, df = 2 (P = 0.01); $I^2 = 78\%$
Test for overall effect: Z = 3.35 (P = 0.0008)

Mean Difference
IV, Random, 95% CI

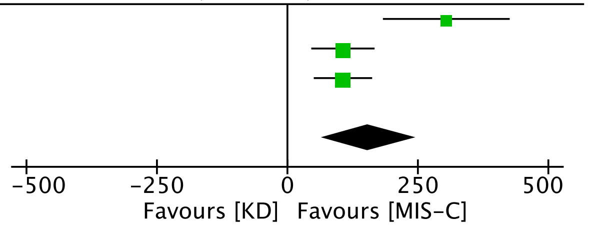

Fig. S5. Troponin in the meta-analysis of six studies using the random-effects model.

Test for overall effect, MD (95% CI): 155.48 (64.54, 246.41), p =0.01

	MIS-C			KD				Mean Difference
**Study or Subgroup**	**Mean**	**SD**	**Total**	**Mean**	**SD**	**Total**	**Weight**	**IV, Random, 95% CI**
Carbajal, R. 2020	20.2	2.2	7	28.1	4.5	40	15.4%	−7.90 [−10.04, −5.76]
Cattalini, M. 2021	30.061	6.8583	53	32.9439	6.774	96	15.3%	−2.88 [−5.17, −0.59]
Consiglio, C. R. 2020	23.744	14.4517	13	36.643	3.906	28	10.4%	−12.90 [−20.89, −4.91]
Esteve-Sole, A. 2021	34.2343	7.9509	12	34.4322	5.5635	14	12.9%	−0.20 [−5.56, 5.16]
Felsenstein, S. 2021	30.5672	6.1915	34	30.5672	6.1915	27	14.8%	0.00 [−3.13, 3.13]
Kostik, M. M. 2021	30.1062	6.2033	72	38	7.487	147	15.5%	−7.89 [−9.77, −6.02]
Whittaker, E. 2020	24	4.5613	58	38	4.4535	1132	15.8%	−14.00 [−15.20, −12.80]
**Total (95% CI)**			**249**			**1484**	**100.0%**	**−6.46 [−10.82, −2.09]**

Heterogeneity: $Tau^2 = 31.04$; $Chi^2 = 138.45$, df = 6 ($P < 0.00001$); $I^2 = 96\%$
Test for overall effect: $Z = 2.90$ ($P = 0.004$)

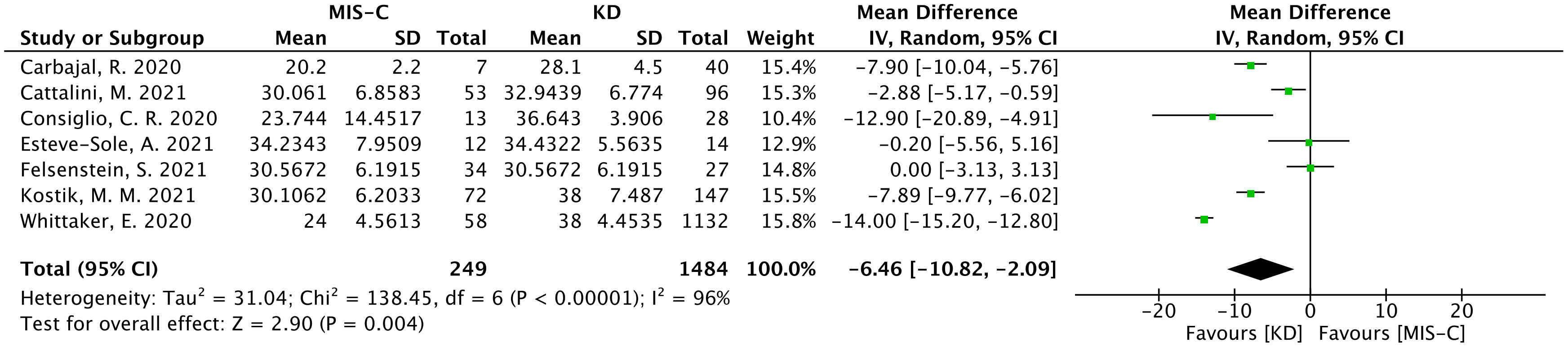

Fig. S6. Albumin in the meta-analysis of six studies using the random-effects model.

Test for overall effect, MD (95% CI): -6.46 (-10.82, -2.09), p =0.004

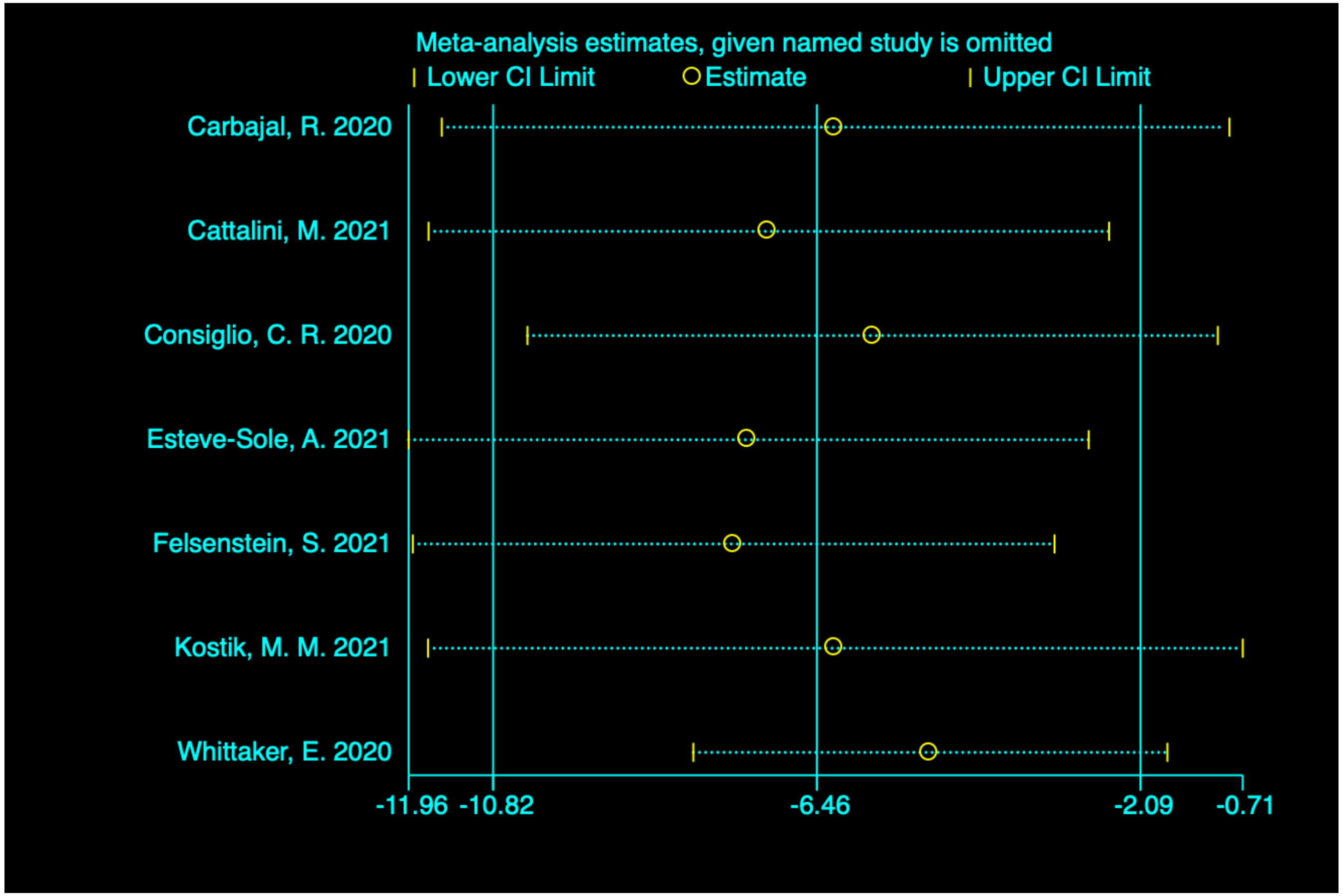

Fig. S7. Sensitivity analysis of albumin

Not any single study was detected to incur undue weight in the analysis.

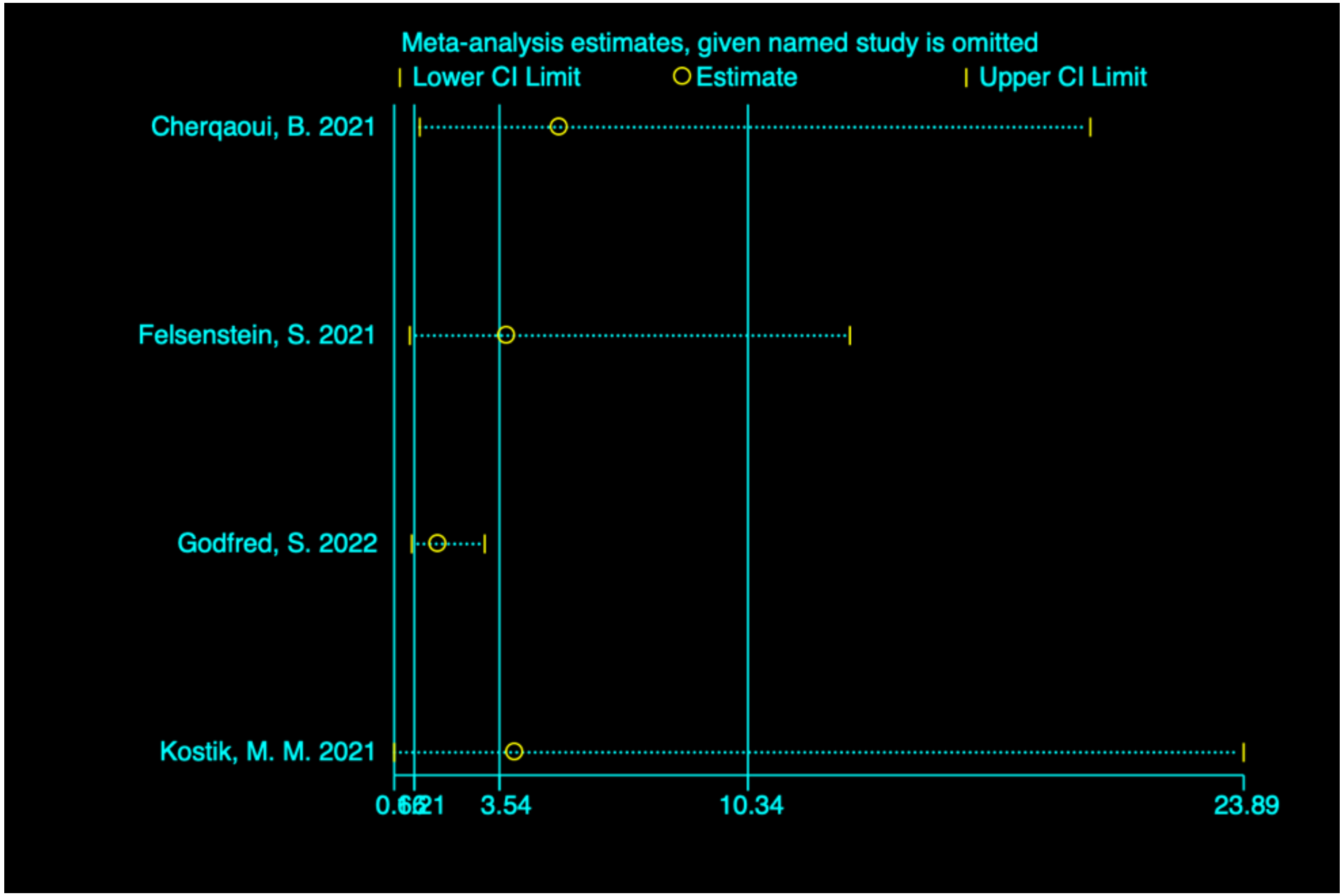

Fig. S8. Sensitivity analysis of neurological symptoms.

When Kostic, M. M.'s study was excluded, the differences were not significant

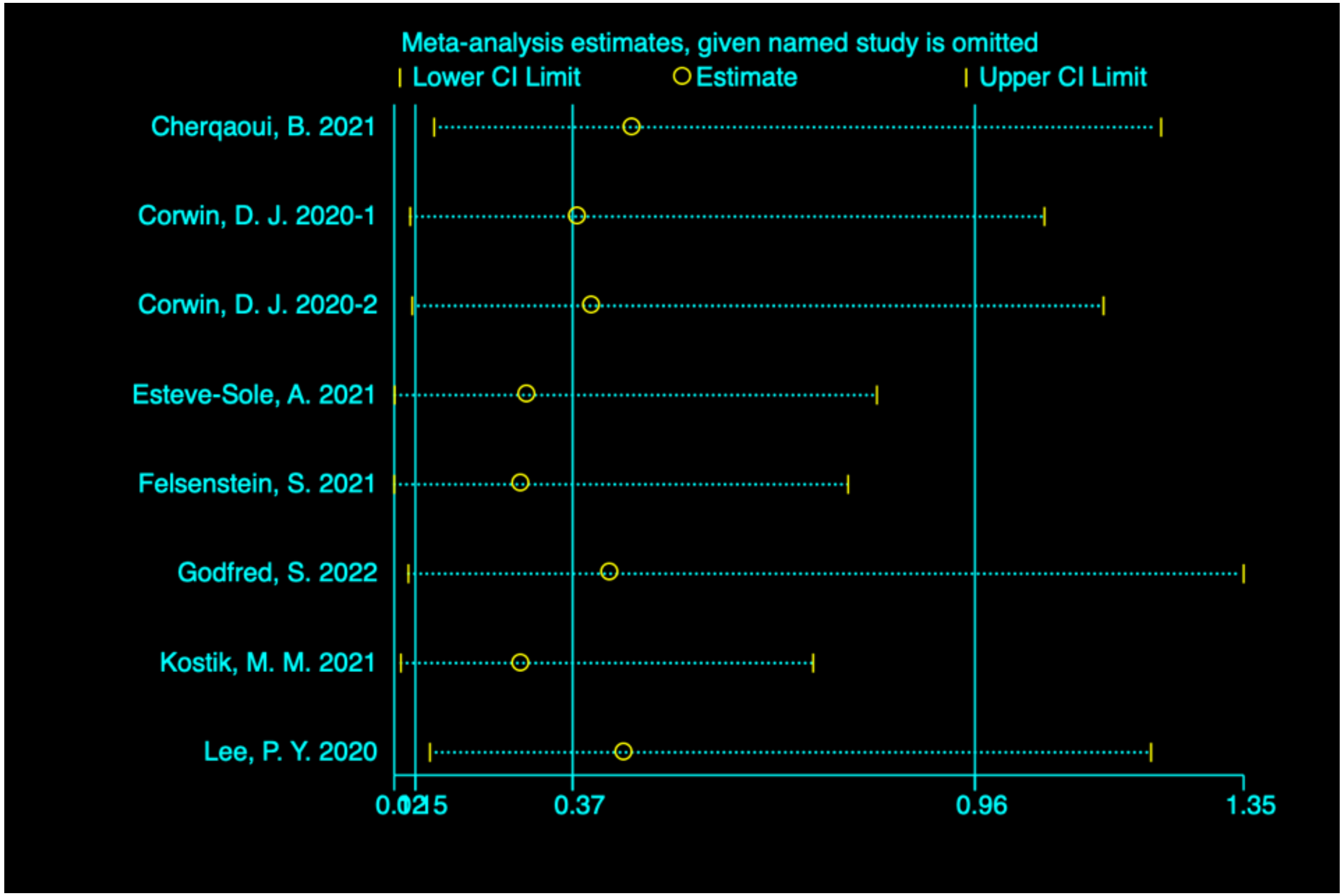

Fig. S9. Sensitivity analysis of cervical lymphadenopathy.

The result was not stable.

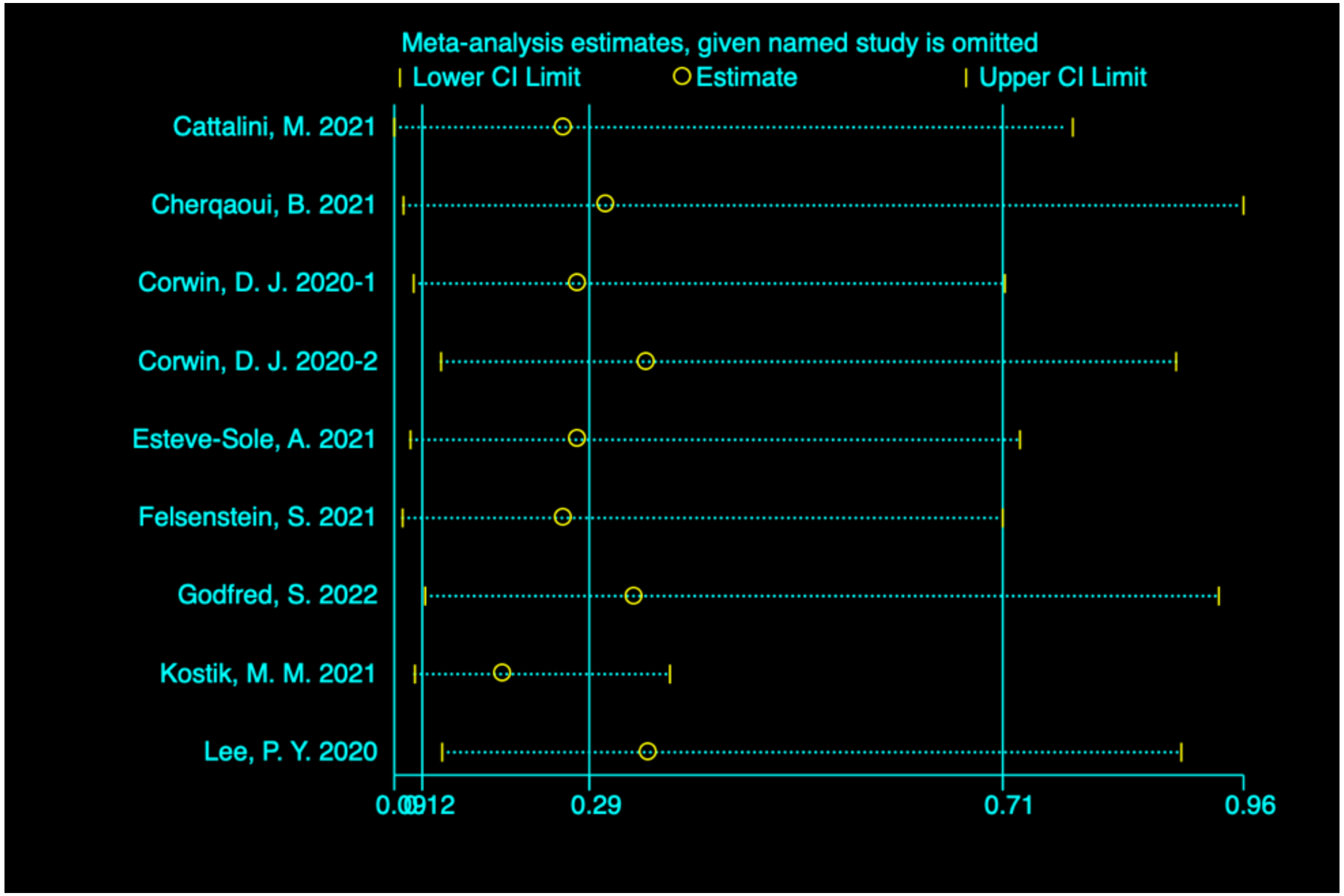

Fig. S10. Sensitivity analysis of conjunctivitis

Not any single study was detected to incur undue weight in the analysis.

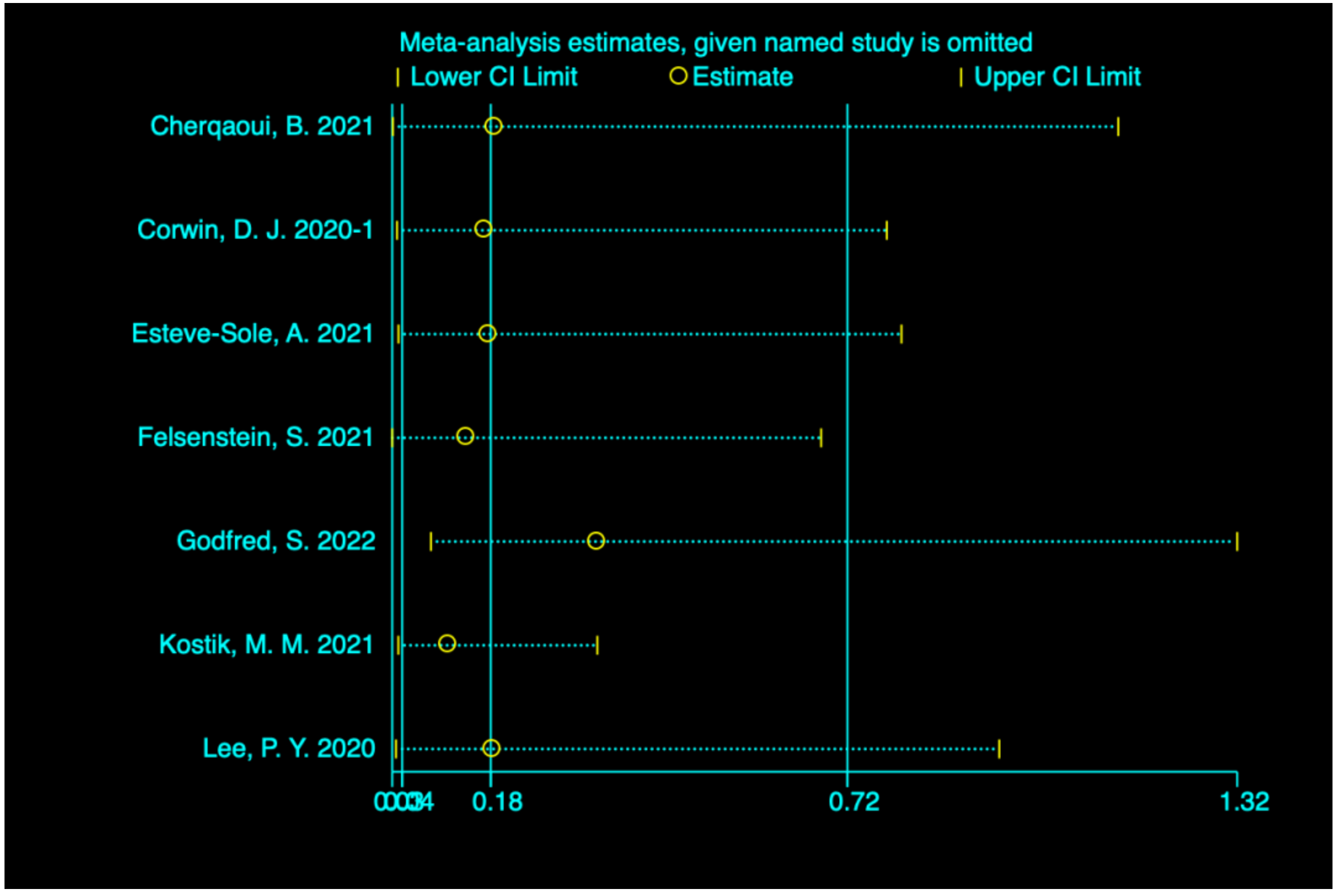

Fig. S11. Sensitivity analysis of oral changes

The result was not stable.

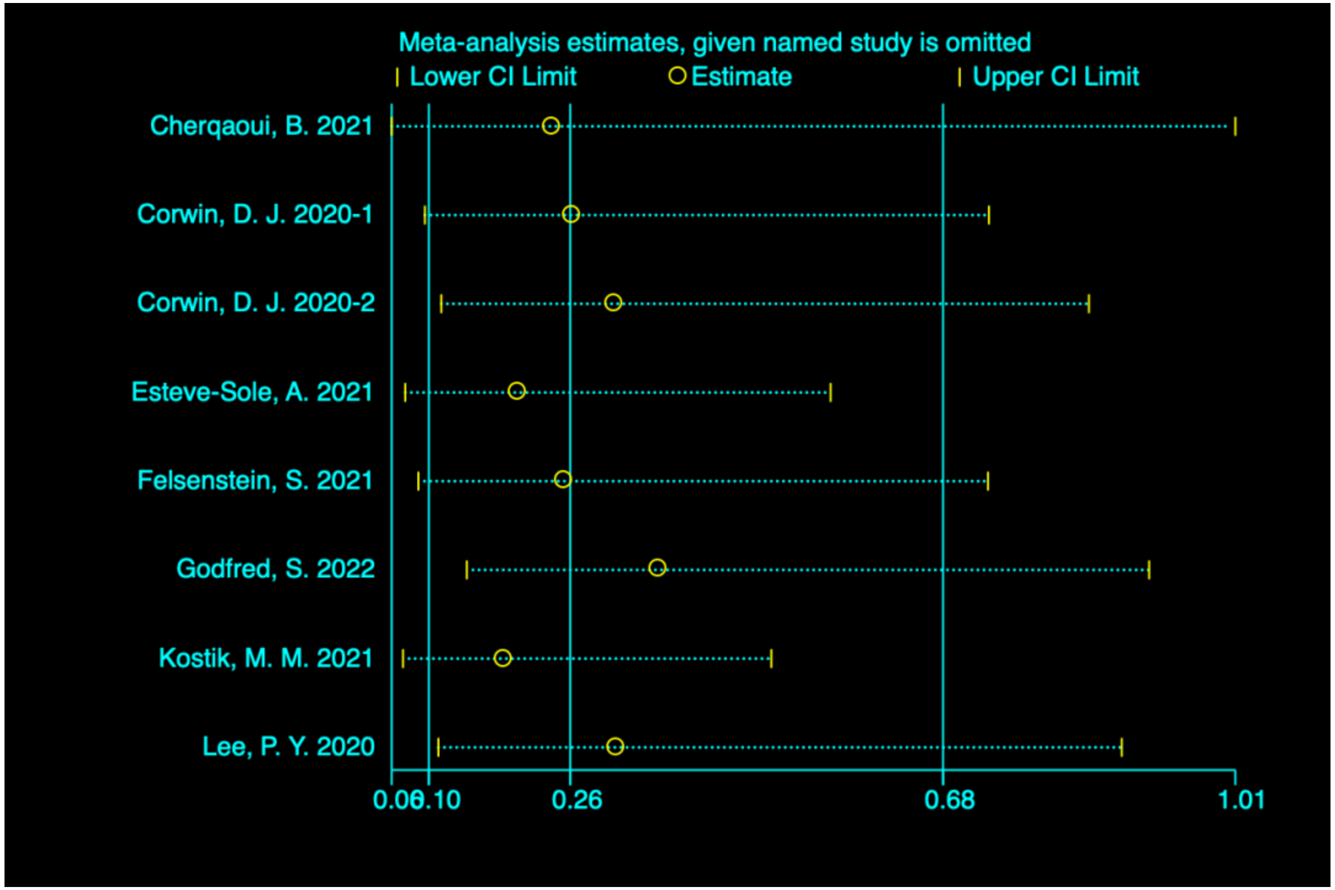

Fig. S12. Sensitivity analysis of rash

The result was not stable.

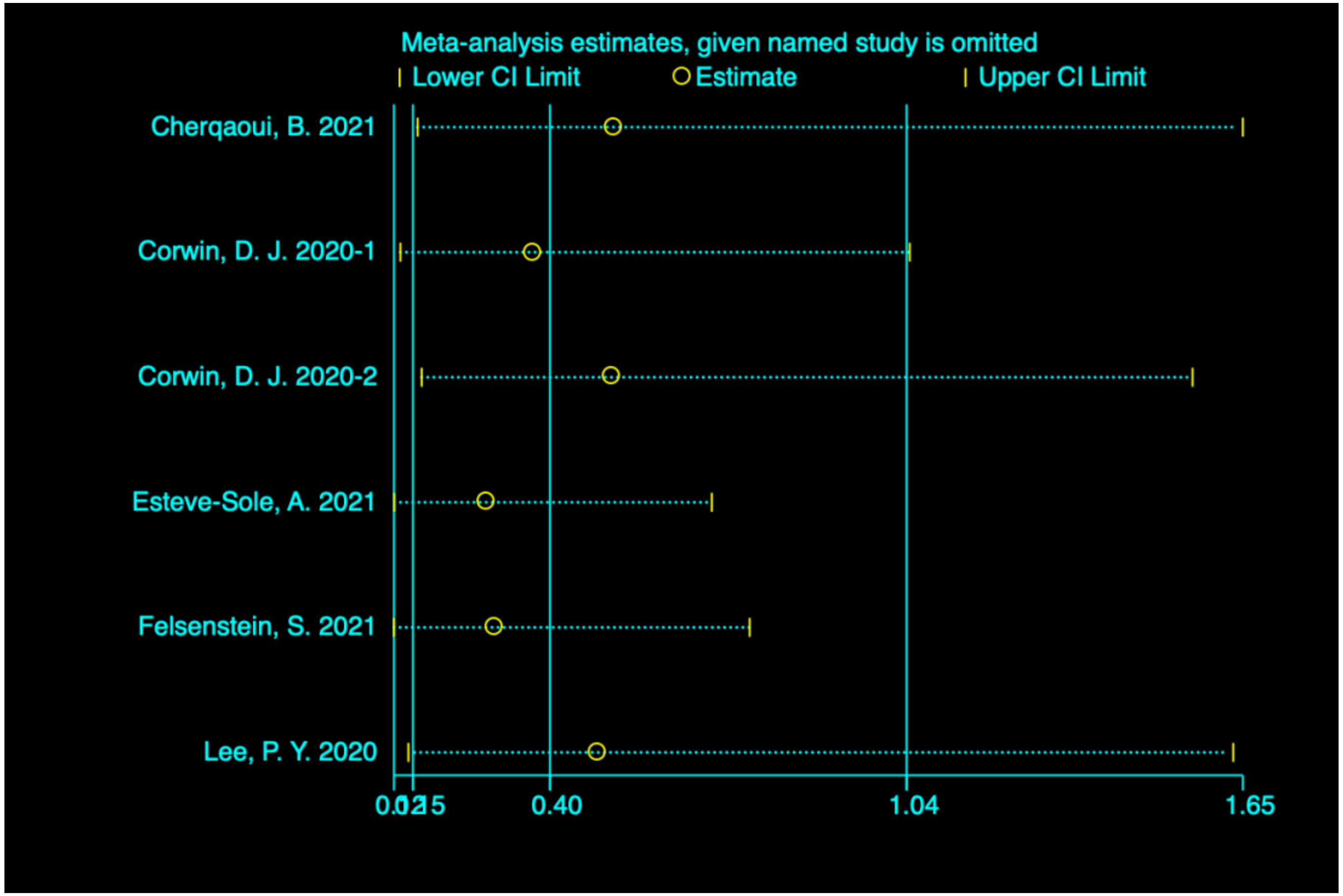

Fig. S13. Sensitivity analysis of extremity changes

The result was not stable.

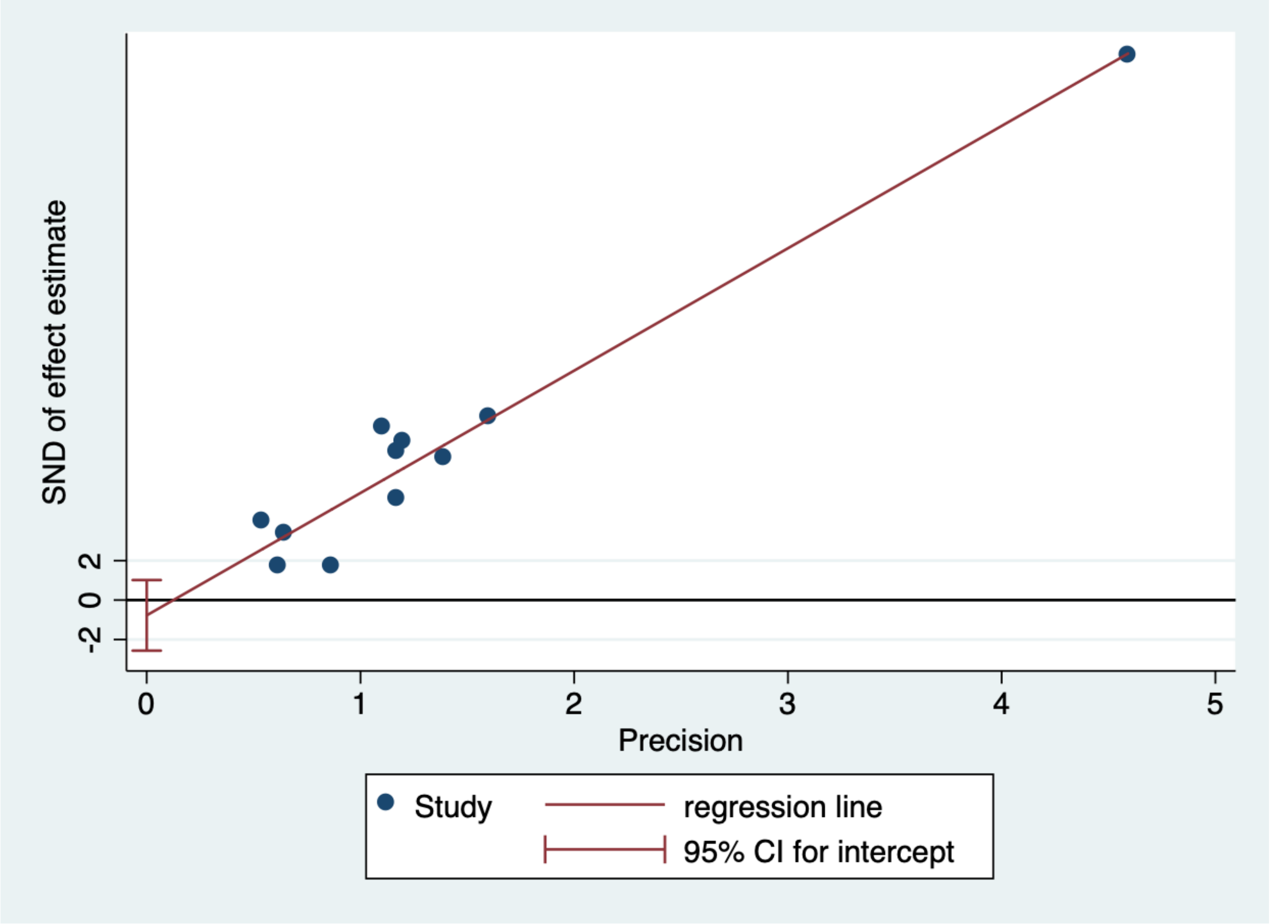

Fig. S14. Egger's publication bias plots for the assessment of potential publication bias in the analysis of age.

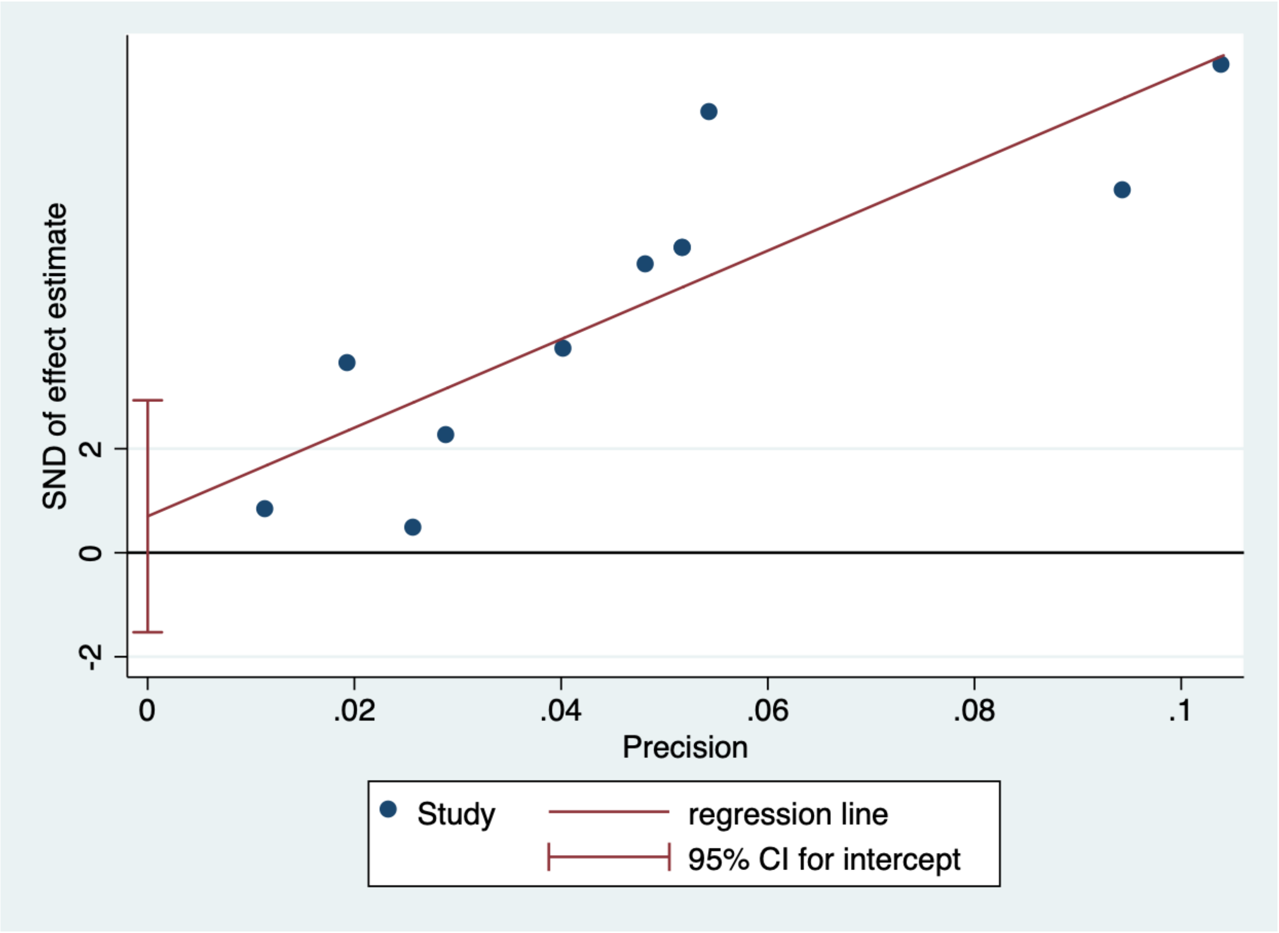

Fig. S15. Egger's publication bias plots for the assessment of potential publication bias in the analysis of CRP.

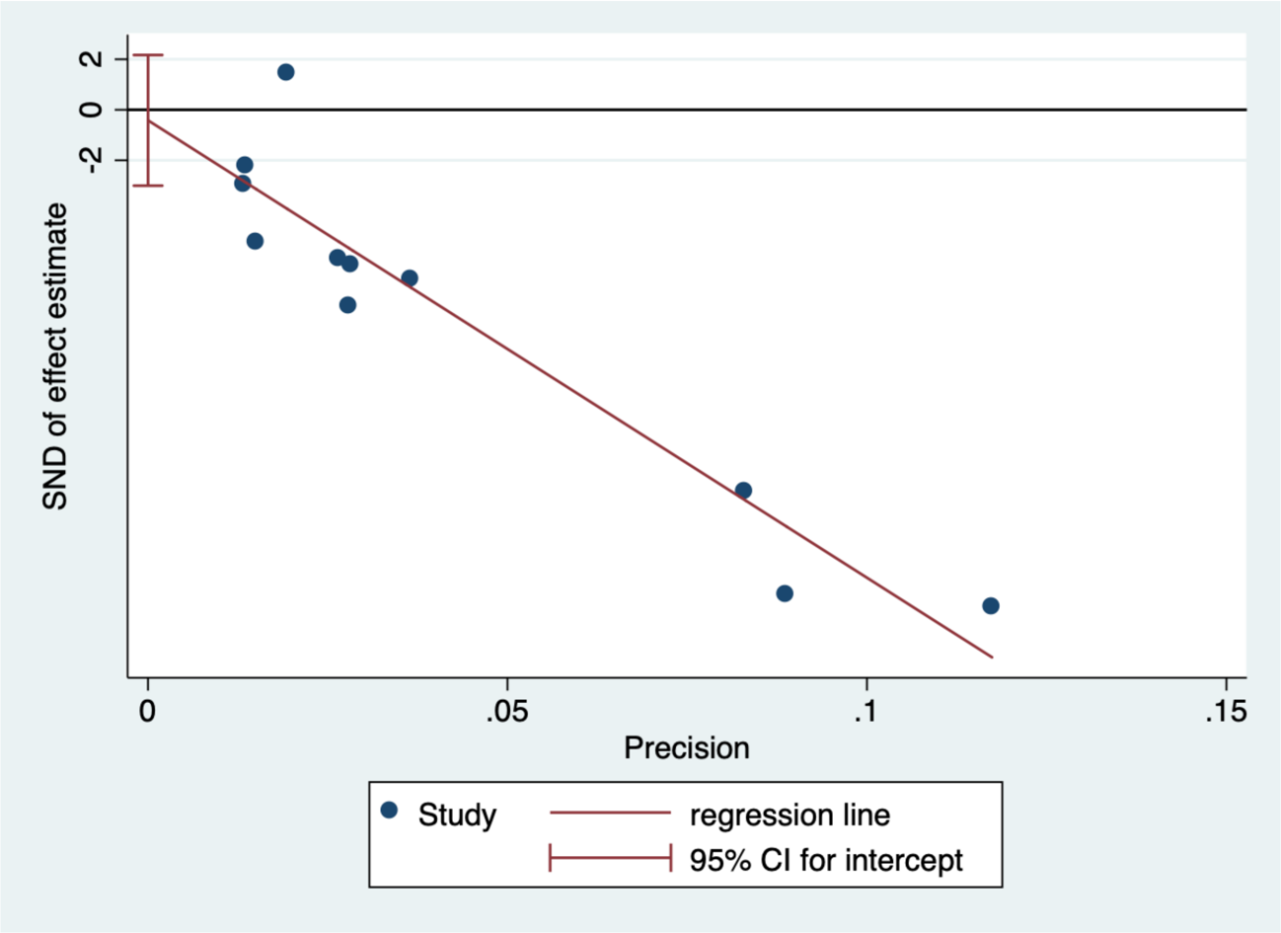

Fig. S16. Egger's publication bias plots for the assessment of potential publication bias in the analysis of platelet count.
